# Ion channel regulation of gut immunity

**DOI:** 10.1085/jgp.202113042

**Published:** 2022-12-02

**Authors:** Jing Feng, Zili Xie, Hongzhen Hu

**Affiliations:** 1 Department of Anesthesiology, The Center for the Study of Itch and Sensory Disorders, Washington University School of Medicine, St. Louis, MO; 2 Center for Neurological and Psychiatric Research and Drug Discovery, Shanghai Institute of Materia Medica, Chinese Academy of Science, Shanghai, China

## Abstract

Mounting evidence indicates that gastrointestinal (GI) homeostasis hinges on communications among many cellular networks including the intestinal epithelium, the immune system, and both intrinsic and extrinsic nerves innervating the gut. The GI tract, especially the colon, is the home base for gut microbiome which dynamically regulates immune function. The gut’s immune system also provides an effective defense against harmful pathogens entering the GI tract while maintaining immune homeostasis to avoid exaggerated immune reaction to innocuous food and commensal antigens which are important causes of inflammatory disorders such as coeliac disease and inflammatory bowel diseases (IBD). Various ion channels have been detected in multiple cell types throughout the GI tract. By regulating membrane properties and intracellular biochemical signaling, ion channels play a critical role in synchronized signaling among diverse cellular components in the gut that orchestrates the GI immune response. This work focuses on the role of ion channels in immune cells, non-immune resident cells, and neuroimmune interactions in the gut at the steady state and pathological conditions. Understanding the cellular and molecular basis of ion channel signaling in these immune-related pathways and initial testing of pharmacological intervention will facilitate the development of ion channel–based therapeutic approaches for the treatment of intestinal inflammation.

## Teaser

This Review discusses ion channel expression and function in immune cells, residential non-immune cells and neuroimmune interactions in the gut, and how ion channels contribute to the maintenance of intestinal immune homeostasis and generation of autoimmune disorders under pathological conditions.

## Introduction

The gut is the largest immune organ in the body (Chassaing et al., 2014). Like in the skin and airways, the mucosal surfaces in the gastrointestinal (GI) tract constitute the largest barrier surface in the body. The intestinal mucosal surfaces are lined by intestinal epithelial cells (IECs) that interact with their external environments to regulate nutrient absorption and protection from harmful stimuli in the gut lumen (Turner, 2009). Among these harmful stimuli are pathogens, xenobiotics, and food antigens that have the potential to disrupt the intestinal barrier and subsequently promote systemic inflammation and tissue damage, especially in individuals with genetic and immune predisposition. Besides maintaining intestinal barrier integrity, the IECs also respond to both chemical and physical stimuli in the gut lumen, and subsequently release immunological factors including cytokines and chemokines to regulate host immune responses (Chen et al., 2022).

The gut microbiota actively regulates the integrity and function of the intestinal barrier, and changes in the composition and function of the gut microbiota contribute to intestinal barrier dysfunction (Takiishi et al., 2017). Emerging evidence demonstrates that there is an imbalance in both intestinal microbiota and mucosal immunity as well as intestinal barrier integrity in many autoimmune diseases such as inflammatory bowel diseases (IBD), suggesting that the gut microbiota–immune system–intestinal barrier axis plays a crucial role in controlling gut immune homeostasis and tolerance at the steady state and regulating abnormal autoimmune responses (Antonini et al., 2019). The mucosal immune system comprises both innate immune cells, such as macrophages and DCs and the majority of the body’s lymphocytes population which are embedded throughout the epithelial layers and exposed to the lumen (Kaymak et al., 2021). There is evidence that both innate and adaptive immune cells can contribute to the regulation of the gut microbiota’s biogeographical distribution along the GI tract, suggesting a bi-directional regulation between the gut microbiota and immune system (Gu et al., 2022).

Besides resident and infiltrated cells in the gut epithelium, the intestinal wall is densely innervated by both extrinsic and intrinsic nerves (Phillips and Powley, 2007). Autonomic nervous system (ANS) comprising the sympathetic and parasympathetic nervous systems receives commands from the central nervous system (CNS) and regulates various GI functions (Browning and Travagli, 2014). Primary sensory nerves extensively project to the GI tract and mediate interoception, the perception of sensations from inside the body (Maniscalco and Rinaman, 2018; Bai et al., 2019). Moreover, the GI tract is the only abdominal organ that has evolved with its own nervous system fully contained within the gut wall, known as the enteric nervous system (ENS), also known as the “second brain” in the gut (Furness, 2012; Wood, 2016; Gershon and Margolis, 2021). Interactions between extrinsic and intrinsic nervous system maintain normal GI motility through regulating the GI smooth muscle tone (Kulkarni et al., 2018). In addition to motility regulation, both intrinsic and extrinsic nerves in the gut interact with immune cells in the epithelium and contribute to the immune homeostasis at steady state and autoimmune responses under pathological conditions (Wang et al., 2022).

**Figure 1.**
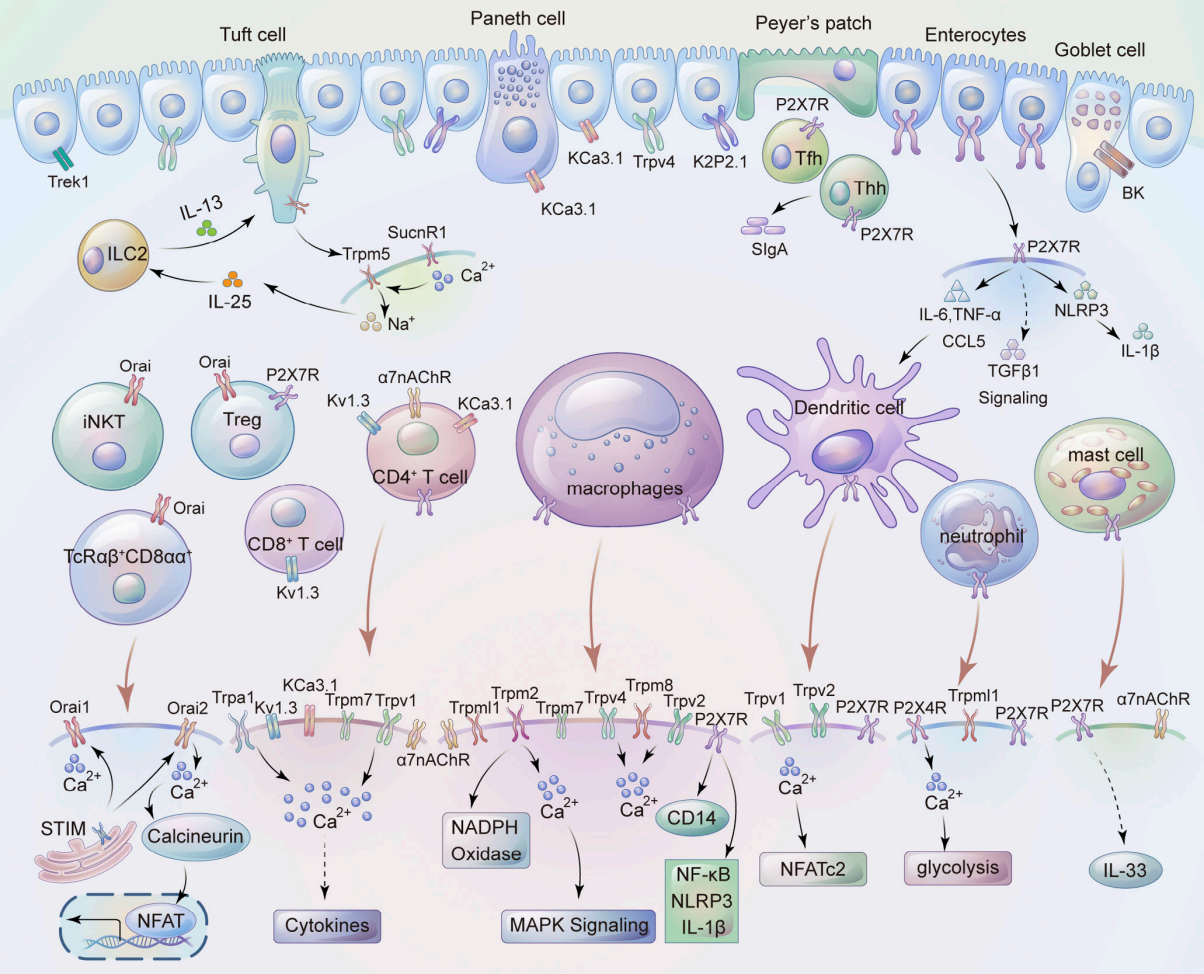
**Summary of cell types and associated ion channels involved in gut immunity based on current literatures.** Distinct cell types present in the intestinal epithelium, such as enterocytes, tuft cell, Paneth cell, Peyer’s patch and goblet cell, sense either internal or external stimuli through potassium channel, TRP channel, and P2X receptors, and maintain the integrity of the intestinal epithelium. Immune cell-expressing ion channels regulate immune activation and cytokine/chemokine release in the intestinal inflammation through provoking Ca^2+^ influx and intracellular Ca^2+^ mobilization.

**Figure 2.**
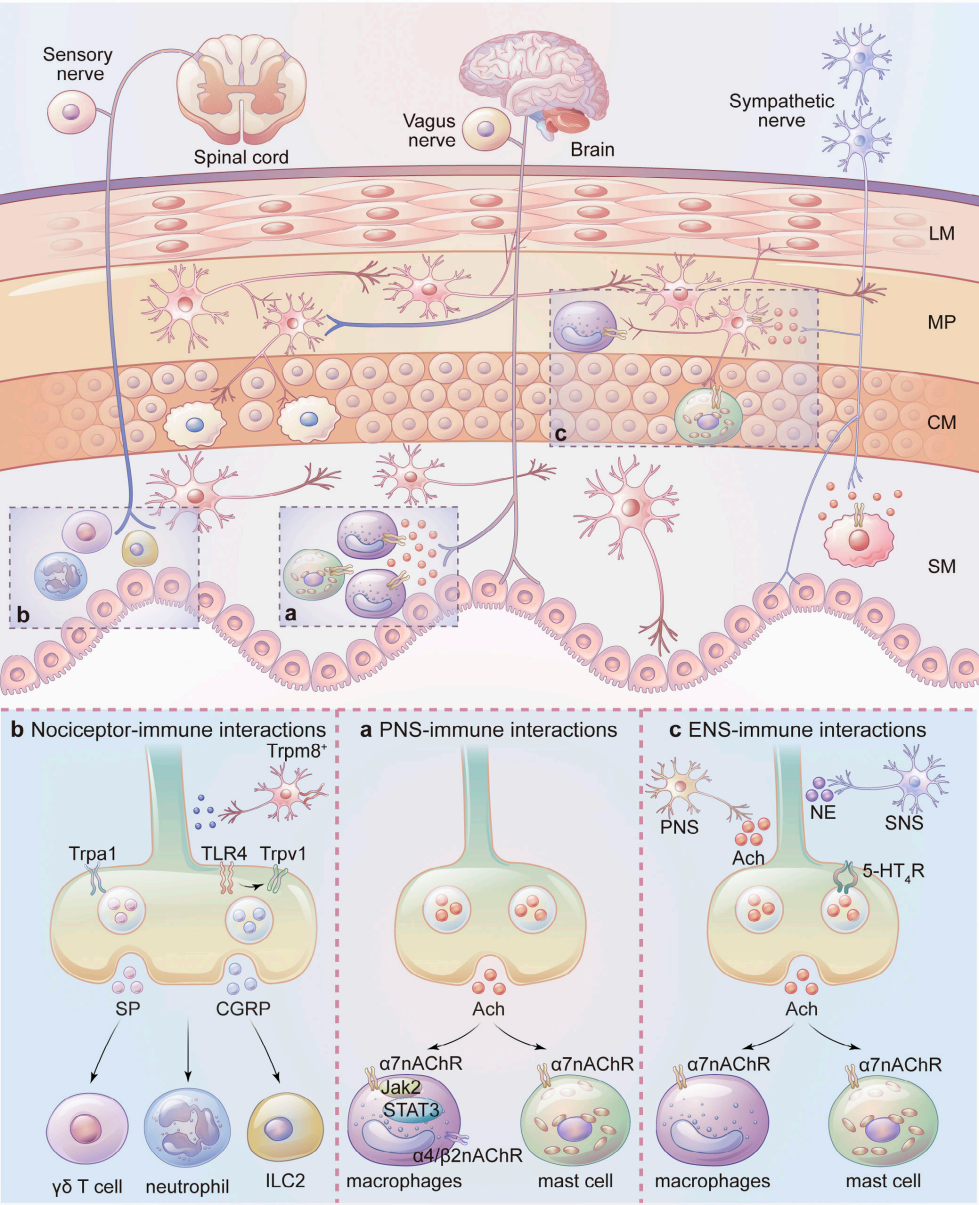
**Ion channels in gut immunity involving crosstalk between gut-innervating neurons and resident immune cells.** The GI tract is innervated by spinal cord sensory neurons, brainstem originating vagus neuron and intrinsic enteric neurons. The VNS-immune interaction (a) is mediated by the release of ACh from the vagus nerve, which suppresses the activation of mast cells and macrophages. TRP channels expressed by nociceptors control the release of neurotransmitters such as SP and CGRP, which may modulate the activities of immune cells and contribute to the nociceptor-immune interaction (b). ENS activation causes acetylcholine release and subsequently modulates the function of monocytes/macrophages in the setting of intestinal inflammation (c). It should be noted that both sympathetic and parasympathetic nerve systems can act indirectly through the ENS. LM, longitudinal muscle; MP, myenteric plexus; CM, circular muscle; SM, submucosa.

Accumulating evidence suggests that ion channels are expressed by both excitatory and non-excitatory cells throughout the GI tract (Fuentes and Christianson, 2016; Fig. 1). Various ion channels are involved in regulating neurotransmission and neurotransmitter release in both intrinsic and extrinsic nerves, ion secretion and absorption in IECs, production and release of cytokines from immune cells, and intestinal smooth muscle contraction (Fuentes and Christianson, 2016; Fig. 2). Channelopathies resulting from abnormal expression and function can often impact GI functions including motility, secretion, visceral pain, immune dysregulation, and gut–brain communications (Beyder and Farrugia, 2016). This Review focuses on recent progresses in understanding the roles of various ion channels in intestinal immune cells, non-immune resident cells, and neuroimmune interactions. Understanding the molecular and cellular mechanisms underlying ion channel regulation of GI immunity will facilitate the identification and development of ion channel-based therapeutic approaches for the treatment of immune disorders in the GI tract.

## Ion channel functions in immune cells in the gut

### Ca^2+^ release-activated Ca^2+^ (CRAC) channels

Ca^2+^ ions are essential to the development, activation, and maintenance of the immune system (Vig and Kinet, 2009). It is well established that both activation and proliferation of T cells require Ca^2+^ entry (Cahalan and Chandy, 2009; Feske et al., 2015). This is nicely reflected by the findings that at steady-state human intestinal lamina propria T cells exhibit minimal proliferation upon stimulation of antigen receptor when compared with T cells in the blood. This low proliferation activity is correlated with reduced Ca^2+^ signaling in isolated lamina propria T cells from non-inflamed tissue. Strikingly, GI inflammation promotes the proliferation of lamina propria T cells and normalizes their Ca^2+^ signaling to the levels in the blood T cells in IBD patients (Schwarz et al., 2004). Moreover, Ca^2+^ influx is required for T-cell proliferation in both peripheral blood and intestinal lamina propria, further highlighting the essential role of Ca^2+^ signaling in T-cell immunity (Schwarz et al., 2004).

The most studied Ca^2+^ entry pathway in T cells is store-operated calcium entry (SOCE) mediated by CRAC channels, which are encoded by the ORAI1 gene and regulated by stromal interaction molecules STIM1 and STIM2 within endoplasmic reticulum (ER)–plasma membrane (PM; Lewis, 2001; Oh-Hora et al., 2013; Vaeth et al., 2020). The pore of the CRAC channel is formed by PM protein Orai1 and Orai2 while stromal interaction molecules (STIM) sense the drop of cytosolic Ca^2+^ concentration and subsequently engage with the Orai proteins to drive Ca^2+^ influx (Vaeth et al., 2020). CRAC channels are the major player to trigger immune responses in T cells, while other ion channels such as K^+^ channels modulate Ca^2+^ signaling through altering membrane potential of T cells to provide the driving force for Ca^2+^ entry (Cahalan and Chandy, 2009). Repetitive or prolonged increase in Ca^2+^ influx through CRACs activates NFAT, thereby promoting T-cell proliferation and cytokine gene expression through the Ca^2+^–calcineurin–NFAT signaling pathway (Park et al., 2020). Interestingly, STIM-induced SOCE is specifically required for the development of agonist-selected T cells (regulatory T cells, invariant natural killer T cells, and TCRαβ^+^ CD8αα^+^ intestinal intraepithelial lymphocytes) but not for thymic development of conventional TCRαβ^+^ T cells, suggesting CRAC-mediated Ca^2+^ signaling regulates maturation of distinct T-cell lineages (Oh-Hora et al., 2013; Fig. 1).

T and B cells from Orai1 knock-in mice (Orai1^KI/KI^) expressing a nonfunctional ORAI1-R93W protein display severely impaired SOCE and CRAC channel function, resulting in a strongly reduced expression of several key cytokines including IL-2, IL-4, IL-17, interferon-γ (IFNγ), and TNFα in CD4^+^ and CD8^+^ T cells. In addition, T cells from the Orai1^KI/KI^ mice fail to develop colitis in an adoptive transfer model of IBD (McCarl et al., 2010). Pharmacological inhibition of SOCE using the CRAC channel inhibitor BTP2 also selectively reduces the production of pathogenic cytokines by human colonic T cells and innate lymphoid cells (ILCs), which alleviates the clinical course of colitis in mice. Moreover, T-cell-specific genetical ablation of Orai1 and Stim1 attenuates the severity of intestinal inflammation in a mouse model of IBD. Most importantly, treatment with a selective CRAC channel inhibitor CM4620 attenuates IBD severity and colitogenic T-cell function in mice (Letizia et al., 2022). These findings reaffirm the requirement of Orai/Stim signaling for T-cell function and provide important insights into the in vivo functions of CRAC channels in the immune response underlying IBD pathogenesis. Therefore, CRAC channels have been considered as drug targets of T cells in immune-mediated disorders, including IBD. Indeed, a selective CRAC inhibitor Synta 66 (GSK1349571A) inhibits CRAC current in rat basophilic leukemia cells and thapsigargin-induced Ca^2+^ influx in Jurkat T cells in a concentration-dependent manner. When used to treat lamina propria mononuclear cells and biopsy specimens from inflamed areas of patients with IBD, Synta 66 reduces T-bet expression and inflammatory cytokine production (Di Sabatino et al., 2009). Interestingly, urolithin A, one of the urolithins of microbiota-derived metabolites from ellagic acid, can inhibit SOCE in murine CD4^+^ T cells and downregulates the expression of Orai1 and STIM1/2 through enhancing the expression of miR-10a-5p (Zhang et al., 2019; Fig. 1). Despite these promising results in modulating T-cell activation by CRAC inhibitors, no clinical trial has been conducted on any classes of CRAC blockers in IBD patients (Tian et al., 2016; Giuffrida and Di Sabatino, 2020). Besides IBD, CRAC channels are also involved in *helicobacter pylori* (*H. pylori*)-induced GI pathology (Kern et al., 2015). *H. pylori* secrets vacuolating toxin (VacA), which is colocalized with STIM1 in the ER and may restrict STIM1 interaction with membrane bound Orai1 upon Ca^2+^ store depletion, thereby inhibiting T-cell activation and proliferation and suppressing host immune response (Kern et al., 2015). Moreover, SOCE is essential for the cytotoxic function of cytotoxic T lymphocytes (CTLs) both in vivo and in vitro by regulating the degranulation of CTLs, their expression of Fas ligand and production of TNFα and IFNγ. The presence of SOCE in CTLs can prevent the engraftment of colon carcinoma cells and control tumor growth. In contrast, patients with loss-of-function mutations in either Orai1 or STIM1 are immunodeficient and are prone to develop virus-associated tumors (Weidinger et al., 2013; Fig. 1).

### Transient receptor potential (TRP) channels

TRP channels constitute a superfamily of Ca^2+^-permeable, non-selective cation channels (Wu et al., 2010). Among TRP channels, TRPM4 and TRPM5 are permeable to monovalent cations but impermeable to divalent cations. Although other TRP channels are Ca^2+^ permeable, the selectivity varies greatly due to their distinct pore structures. For instance, P_Ca_/P_Na_ ratio is <1 for TRPM1 but >100 for TRPV5 and TRPV6 (Gees et al., 2010; Yelshanskaya et al., 2021). TRP channels are traditionally considered as molecular sensors for temperature, pain, itch, vision, sound, and taste; however, recent studies indicated that they may also be involved in various other physiological functions including immune regulation (Inada et al., 2006; Parenti et al., 2016; Froghi et al., 2021). Both innate and adaptive immune cells express various types of TRP channels which actively regulate immune response primarily by activating intracellular Ca^2+^ responses and related intracellular-signaling pathways, thereby affecting immune cell proliferation/differentiation, cytokine production, and cellular cytotoxicity (Inada et al., 2006; Parenti et al., 2016; Froghi et al., 2021). Not surprisingly, several TRP channels are closely related to GI inflammation (Parenti et al., 2016; Froghi et al., 2021).

### TRPA1

TRPA1 is primarily expressed by primary nociceptors and serves as a critical mediator of pain and itch sensations (Xie and Hu, 2018). TRPA1 is activated by diverse electrophilic irritants which covalently modify intracellular reactive cysteine residues (Hinman et al., 2006; Macpherson et al., 2007). Activated TRPA1 possesses high Ca^2+^ permeability, while increased intracellular free Ca^2+^ can also actively regulate TRPA1 function through an intracellular Ca^2+^-binding pocket (Zhao et al., 2020). Although TRPA1 along with the capsaicin receptor TRPV1 is exclusively expressed by the dorsal root ganglion (DRG) neurons, recent studies raised the possibility that TRPV1 and TRPA1 could be functionally expressed by the intestinal CD4^+^ T cells, although with opposite functions: TRPV1 is shown to be related to TCR-mediated Ca^2+^ influx, T-cell activation, and differentiation into Th1-effector cells, while TRPA1 is reported to modulate these pro-inflammatory T-cell responses (Bertin et al., 2014; Bertin et al., 2017). This functional difference is also reflected by a TRPV1-mediated promotion but a TRPA1-mediated inhibition of experimental colitis in mice (Bertin et al., 2014; Bertin et al., 2017). Moreover, TRPA1 expression is upregulated in inflamed colon tissues from both humans and mice (Kun et al., 2014; Bertin et al., 2017; Fig. 1). It is intriguing that although both TRPV1 and TRPA1 mediate Ca^2+^ influx that initiates intracellular Ca^2+^ signaling in a similar manner, loss of function of TRPV1 and TRPA1 show opposite phenotypes in mouse models of experimental colitis (Bertin et al., 2014; Bertin et al., 2017). These contradictory findings suggest that TRP channel action in GI immunity may be more complicated than we thought. Moreover, although it was shown that TRPV1 and TRPA1 function in CD4^+^ T cells (Bertin et al., 2014; Bertin et al., 2017), mRNA encoding these channels are barely detectable according to available gene expression databases (such as https://www.immgen.org or https://biogps.org). It should also be noted that these functional studies are dependent on the use of global knockout (KO) mice. Since both TRPV1 and TRPA1 are expressed by primary nociceptors, the potential involvement of neuroimmune interactions in the setting of chemical-induced intestinal inflammation cannot be excluded. Future studies with immune cell-specific TRPA1 and TRPV1 KO mice should be employed to further support the role of TRP channels in gut immunity.

### TRPM2

TRPM2 is a Ca^2+^-permeable channel that activated by warm temperatures, oxidative stress, and NAD^+^-related metabolites such as ADP-ribose (ADPR; Huang et al., 2018). TRPM2 is functionally expressed by macrophages and TRPM2-deficient mice have enhanced gastric inflammation and decreased bacterial colonization when chronically infected with *H. pylori*, compared with wild-type mice. This is because TRPM2-deficient macrophages are unable to control intracellular Ca^2+^ levels, resulting in Ca^2+^ overload which subsequently enhances activities of both mitogen-activated protein kinase (MAPK) and NADPH-oxidase upon *H. pylori* stimulation (Beceiro et al., 2017). In a mouse model of dextran sulphate sodium (DSS)-induced colitis, TRPM2 deficiency reduces the production of the CXCL2 chemokine, resulting in diminished neutrophilic influx to the colon, and protects against colon inflammation, suggesting that pharmacological inhibition of TRPM2 might be a new therapeutic strategy for treating IBD (Yamamoto et al., 2008; Knowles et al., 2013; Fig. 1).

### TRPM7

TRPM7 chanzyme harbors both a cation channel and a serine/threonine kinase and has been implicated in regulating thymopoiesis and cytokine expression. Silencing TRPM7 function promotes the development of thymic Treg cells through the downstream IL-2–IL-2R and FOPX3 signaling (Mendu et al., 2020). TRPM7 kinase activity also contributes to TGF-β–induced CD103 expression and pro-inflammatory Th17 cell differentiation and colonization in the gut (Romagnani et al., 2017). Interestingly, loss of TRPM7-mediated cation influx (such as Ca^2+^ and Mg^2+^), but not its kinase activity, was shown to prevent LPS-TLR4 signaling-induced macrophage activation (Schappe et al., 2018), increase apoptosis of B cell precursors, and significantly reduce the number peripheral B cells (Krishnamoorthy et al., 2018). Surprisingly, TRPM7 kinase-dead (KD) K1646R knock-in mice, which have a similar TRPM7-mediated currents with wild-type T cells, show reduced store-operated Ca^2+^ entry in T lymphocytes, resulting in reduced T-cell proliferation and enlarged spleen in mice (Beesetty et al., 2018). Although rare evidence was shown that TRPM7 is involved in the intestinal homeostasis, a possibility was raised that TRPM7 may participate in both IBD-related and sporadic colorectal cancer, as TRPM7 is overexpressed in human IBD-related and sporadic colorectal cancer (Pugliese et al., 2020). Future studies on the role of TRPM7 in the GI tract may shed new light on the dual functions of TRPM7 chanzyme in gut immunity.

### TRPM8

TRPM8 is expressed by primary sensory neurons playing a central role in detecting environmental cold temperatures and natural compounds including menthol (McKemy et al., 2002). Unexpectedly, with in vitro molecular techniques and Ca^2+^ imaging assay, TRPM8 expression and function were also detected in both peritoneal and bone marrow-derived macrophages (Khalil et al., 2016). TRPM8 is required for macrophage phagocytosis and maintaining an anti-inflammatory cytokine profile (Khalil et al., 2016). Pharmacological activation of TRPM8 by repeated applications of menthol enemas protects against DSS-induced colitis while global TRPM8 KO aggravates colitis severity, which is correlated with the profile of macrophage-derived TNFα (inflammatory) and interleukin-10 (anti-inflammatory) cytokine production (Khalil et al., 2016; Fig. 1). However, these in vivo data were generated from global TRPM8 KO mice, which could not exclude the possibility that the TRPM8 expressed by sensory neurons may modulate the macrophage functions through neuroimmune crosstalk. Conditional immune cell-specific TRPM8 KO mice are needed to validate the role of TRPM8 in immunomodulation in the gut.

### TRPV1 and TRPV2

Although nociceptor-expressed TRPV1 is essential to the generation of inflammatory thermal pain (Julius, 2013), CD4^+^ T cells were also reported to express functional TRPV1 which was shown to play a pro-inflammatory role in a mouse model of colitis (Bertin et al., 2014), which is in marked contrast to TRPA1 activation of which was shown to attenuate colitis (Bertin et al., 2017). Notably, pharmacological inhibition of TRPV1 using BCTC also restrains pro-inflammatory T-cell responses (Bertin et al., 2014). However, functional expression of TRPV1 in CD4^+^ T cells needs to be further validated as capsaicin does not seem to activate significant whole-cell currents and intracellular Ca^2+^ responses in normal CD4^+^ T cells (Bertin et al., 2014) but evokes robust activation in sensory neurons (Stein et al., 2006). Moreover, the use of high concentrations of capsaicin is a concern as capsaicin activates native TRPV1 in sensory neurons with an EC_50_ around 50 nM (Stein et al., 2006) while 16 μM capsaicin is used to stimulate CD4^+^ T cells (Bertin et al., 2014). It should also be noted that capsaicin can alter membrane ion flux independent of TRPV1 function (Braga Ferreira et al., 2020) and a recent report showed no expression of TRPV1 protein in CD4^+^ T cells in the skin (Kemeny et al., 2018).

Besides CD4^+^ T cells, TRPV1 function was also reported in dendritic cells (DCs) as gain of function of TRPV1 promotes DC activation, enhances activation of NFATc2 signaling, and promotes cytokine production induced by inflammatory stimuli in in vitro tests (Duo et al., 2020). In vivo, TRPV1 gain of function causes excessive recruitment of DCs and enhances Th17 immune responses in lamina propria of colon, thereby increasing the susceptibility of mice to experimental colitis (Duo et al., 2020). However, no TRPV1-mediated membrane currents or intracellular Ca^2+^ responses have been recorded on DCs and an indirect action through neuroimmune interactions cannot be excluded in the gain of function mutants either. Genetic ablation of TRPV2 function was shown to attenuate DSS-induced colitis at the clinical, histopathological and immunohistochemical levels. This result is likely caused by a reduction of macrophage recruitment to the inflammation site (Issa et al., 2014), which is consistent with a report that TRPV2 engages in macrophage particle binding and phagocytosis (Link et al., 2010). These studies suggest that immune cells might possess intrinsic functions of TRPV2 (Fig. 1).

### TRPML1

TRPML1 is predominantly localized on the membranes of late endosomes and lysosomes (LELs). Functionally, TRPML1 mediates the release of Ca^2+^ and heavy metal Fe^2+^/Zn^2+^ ions into the cytosol from the LEL lumen, thus regulating the ingestion of large particles by promoting the maturation of phagosome (Dayam et al., 2015; Dayam et al., 2017) and providing the membrane for the cell surface (Samie et al., 2013), suggesting a potential role of TRPML1 in macrophage/neutrophil phagocytosis. Moreover, inhibition of TRPML1-mediated Ca^2+^ signaling in endolysosome reduces the transportation of single-stranded RNA (ssRNA) into lysosomes in TLR-mediated response to pathogens while chemical activation of TRPML1 by the TRPML channel agonist ML-SA1 enhances this process (Li et al., 2015).

### P2X7 receptor

Extracellular adenosine triphosphate (ATP) is an important intercellular signaling molecule that binds to both ionotropic P2X receptors and G-protein-coupled P2Y receptors. Both P2X and P2Y receptors are widely distributed through the nervous, cardiovascular, and immune systems, and play critical roles in synaptic transmission, somatosensation, and inflammation. Among them, P2X7 purinergic receptor (P2X7R), which mediates transmembrane K^+^ efflux and Ca^2+^ influx in immune cells, engages with extracellular ATP released by mucosal immune cells and microbiota in the gut and is critically involved in intestinal homeostasis through eliciting diverse immune responses (Cheng et al., 2021). Besides P2X7R, genetic manipulation of neutrophil-expressing P2X4R showed that the ATP–P2X4R signaling axis in myeloid cells causes Ca^2+^ influx that promotes glycolysis in response to an increase in microbiota-derived luminal ATP, thereby regulating intestinal inflammation (Tani et al., 2021; Fig. 1).

P2X7R is expressed by both lymphocytes and macrophages as well as IECs in the small bowel although these cells displayed different sensitivity to ATP (de Campos et al., 2012). In colonoscopy samples from Crohn’s disease (CD) patients, P2X7R expression is upregulated primarily in DCs and macrophages, which is correlated with enhanced tissue inflammation and apoptosis. Moreover, autocrine activation of P2X receptors by Pannexin-1–mediated ATP release stimulates IL-2 expression and T-cell proliferation and exacerbates inflammation in murine models of type 1 diabetes and IBD (Schenk et al., 2008). Strikingly, P2X7R KO animals do not develop colitis in response to TNBS, DSS, or oxazolone, suggesting that P2X7R signaling is an important component in the pathogenesis of chemical-induced intestinal inflammation (Neves et al., 2014; Figliuolo et al., 2017). Mechanistically, P2X7R activation triggers regulatory T-cell death and inhibits regulatory T-cell migration to the colon. Pharmacological inhibition or genetic ablation of the P2X7R function increases survival and migration of regulatory T cells which promotes immune system tolerance in the gut (Figliuolo et al., 2017; Fig. 1). Consistent with genetic ablation results, inhibition of P2X7R by orally administered Brilliant blue G or MgCl_2_ attenuates the severity of DSS-induced colitis, which is correlated with decreased accumulation of P2X7R^+^ mast cells in the colon (Ohbori et al., 2017). Moreover, both in vitro and in vivo data showed that the pro-inflammatory cytokine IL-6 increases ATP synthesis and P2X7R-mediated signaling in Tregs, resulting in the conversion of Tregs to Th17 cells, suggesting that inhibition of P2X7R in Tregs may ameliorate tissue inflammation (Schenk et al., 2011). Accordingly, pretreatment with P2X7R inhibitor A740003 was shown to prevents rats from developing TNBS-induced colitis while intraperitoneal administration of A740003 or brilliant blue G is effective in attenuating the severity of TNBS-induced colitis, and reducing myeloperoxidase (MPO) activity, collagen deposition, densities of lamina propria T-cells and macrophages (Marques et al., 2014; Fig. 1).

Interestingly, deficiency in ATP–P2X7R signaling could also disrupt mucosal immune system, leading to intestinal inflammation. For instance, in Peyer’s patches, the organized lymphoid follicles in the intestinal mucus membrane, P2X7R function was shown to control the number of T follicular helper cells (Tfh) that secret high-affinity secretory IgA (SIgA) which binds and depletes mucosal bacteria, resulting in reduced mucosal colonization of commensals (Proietti et al., 2014). P2X7R deficiency in Tfh cells enhances germinal center reactions to produce more Tfh cells to actively eliminate commensals. Since mucosal commensals are critical in shaping host immune system, P2X7R-deficient mice are more susceptible to polymicrobial sepsis (Proietti et al., 2014). Besides controlling the production of SIgA in the Peyer’s patches, P2X7R signaling also helps to maintain elevated concentrations of circulating CD14 during infection to clear sepsis (Alarcon-Vila et al., 2020; Fig. 1). Since both over activation and deficiency of P2X7R can disrupt intestinal immunity, it is critical to selectively manipulate the P2X7R function in the settings of distinct intestinal immune disorders.

P2X7R deficiency in macrophages in the GI tract also increases the worm burdens in murine *Trichinella spiralis* infection (Guan et al., 2021). Mechanistically, *T. spiralis* infection upregulates the expression of P2X7R which subsequently activates the NF-κB/NLRP3/IL-1β pathway in macrophages, which initiates an innate immune program to protect the host from *T. spiralis* infection by increasing the capacity of macrophages to kill newborn *T. spiralis* larvae (Guan et al., 2021). A recent study also showed that P2X7R mediates the activation of mast cells by ATP released from apoptotic IECs upon infection by *Heligmosomoides polygyrus* (Hp) nematode in mice lack Spi-B, the Ets transcription factor regulating myeloid differentiation (Shimokawa et al., 2017). Activated mast cells produce IL-33 which drives the production and activation of the IL-13–producing group 2 innate lymphoid cells (ILC2), which subsequently increases the number of goblet cells to produce mucin to help clearance of Hp infection at the early phase (Shimokawa et al., 2017; Fig. 1).

Besides regulating intestinal inflammation and parasitic infection, P2X7R-mediated signaling also suppresses the development of colitis-associated cancer (CAC; Hofman et al., 2015). In loss of function studies using both genetic and pharmacologic tools, blockade of P2X7R function increases proliferation of IECs and protected them from apoptosis through enhanced TGFβ1 signaling. Moreover, P2X7R blockade also alters immune cell infiltration and promotes Treg accumulation within lesions in the intestines (Hofman et al., 2015). Like other ion channels, specificity is one of the critical issues regarding genetic and pharmacological manipulations of P2X7R. As P2X7R is expressed by diverse cell types, global P2X7R KO approach does not have cellular specificity. Some of the commonly used P2X7R inhibitors such as Brilliant blue G and MgCl_2_ likely also have many off-target actions when given in vivo. Recently, some potent P2X7R antagonists have been developed by several pharmaceutical companies. For example, A-438079 is very appealing for its efficacy in reverting allodynia in the neuropathic pain model. Nevertheless, it has short half-life and low bioavailability (Letavic et al., 2017). JNJ-47965567, which is one of the most used ligands in basic and preclinical research, displays poor oral bioavailability (Bhattacharya et al., 2013). Although high throughput screening identified AZD9056 as a selective orally active inhibitor of P2X7R, it lacks effect on inflammatory biomarkers in a phase IIa study assessing its potential in the treatment of moderately to severely active CD (Eser et al., 2015). Moreover, there are many types of purinergic receptors that can be activated by extracellular ATP and might also be involved in extracellular ATP-induced immune regulation besides P2X7R. Thus, although P2X7R is becoming an exciting target for immune cell-related chronic inflammatory disorders, these potential complications need be taken into consideration in experimental data interpretation and clinical trial design.

### nAChR

Nicotinic acetylcholine receptors (nAChRs) are cholinergic receptors that form ligand-gated ion channels. Both α7 nicotinic acetylcholine receptor (α7nAChR) and α9nAChR subunits were identified in macrophages and B cells (Wang et al., 2003; Zhang et al., 2020). α7nAChR subunit is also expressed by human lamina propria T cells. Chronic nicotine treatment in cell cultures only upregulates the expression of T-bet mRNA (Th1-dominant) in human lamina propria T cells but shows negligible effect on the expression of GATA-3 mRNA (Th2-dominant) through α7nAChRs (Kikuchi et al., 2008), which might explain why chronic nicotine stimulation, such as smoking, modulates immune response of mucosal T cells in IBD, and nicotine has a beneficial influence on ulcerative colitis (UC; Th1-dominant) but not CD (Th2-dominant; Fig. 1).

The α7nAChR protein is also markedly increased in the inflamed colon in a mouse model of DSS-induced colitis and demonstrates a protective role in DSS-induced inflammation as the α7nAChR agonist PNU282987 suppresses DSS-induced colon inflammation in vivo and reduces LPS-induced increase in cytokine production and ROS levels in α7nAChR-expressing macrophages in vitro (Xiao et al., 2020). Similarly, when systemically applied, another α7nAChR partial agonist encenicline attenuates the severity of TNBS- and DSS-induced colitis in mice through the α7nAChRs. Mechanistically, encenicline increases the number of FoxP3^+^ regulatory T cells (immunosuppressive) but reduces the number of IL-17A–producing T cells (pro-inflammatory) in DSS-treated mice, suggesting that activation of α7nAChR by encenicline alleviates colitis through altering the balance of immunosuppressive/pro-inflammatory T cells in the gut (Salaga et al., 2016).

Interestingly, chemical activation of the α7nAChRs expressed by the gastric muscular macrophages of Parkinson’s disease rats by NU-282987 or GTS-21 reduces the number of macrophages, expression of pro-inflammatory mediator, and improves gastric motility (Zhou et al., 2021). GTS-21 also reduces the levels of IL-6 in blood and colon, enhanced colonic permeability, and inflammation in mice subjected to cecal ligation and puncture. In marked contrast, splenectomized animals and α7nAChR KO mice display a more severe septic phenotype, which can also be improved by GTS-21 (Nullens et al., 2016). Although these findings support that direct chemical stimulation of the α7nAChRs is effective in attenuating GI inflammation, Snoek et al. (2010) reported that systemic applications of nicotine and two selective α7nAChR agonists AR-R17779 and GSK1345038A have no significant beneficiary effect on disease severity in two mouse models of experimental colitis. Instead, treatment with AR-R17779 or GSK1345038A worsens disease severity and increases pro-inflammatory cytokine levels in the colon in DSS colitis. These conflicting experimental results regarding the functions of α7nAChR need to be addressed before conclusions are drawn for the role of nAChRs in gut immunity.

Besides expression in T cells and macrophages, nAChRs are also expressed by mucosal mast cells (MMCs) and play an essential role in food allergy (FA). For instance, in a mouse model of FA associated with severe allergic diarrhea, both exaggerated Th2 cell-mediated immune responses and MMC hyperplasia take place in the colon. Vagal stimulation by 2-deoxy-D-glucose and application of nAChR agonist nicotine or GTS-21 is sufficient to attenuate the allergic symptoms in the FA mice by inhibiting MMC hyperplasia and T-cell-mediated immune responses (Yamamoto et al., 2014; Fig. 1). Although emerging evidence supports the involvement of nAChRs in gut immunity, most of these studies rely on pharmacological inhibition and global KO approaches, which do not provide information regarding specific cellular functions. Future studies using cell-specific genetic manipulations are needed to provide more mechanistic studies to validate these findings.

### K^+^ channels

It is well-established that calcium-activated potassium channel KCa3.1 plays an important role in modulating Ca^2+^ signaling through the control of the membrane potential in T lymphocytes (Wulff and Castle, 2010). In the ileum, KCa3.1 expression is primarily detected in Paneth cells and T cells in inflamed lamina propria (Simms et al., 2010). Interestingly, single nucleotide polymorphism mapping has identified KCNN4, a human gene encoding the KCa3.1 protein, as a susceptibility gene in the development of CD in Australian and New Zealand populations (Simms et al., 2010). KCa3.1 mRNA and protein levels were also reported to be upregulated in IECs from CD and UC patients but not in non-IBD intestinal inflammation (Suss et al., 2020). In mouse DSS colitis model, both expression and function of the KCa3.1 channel are upregulated in the CD4^+^ T lymphocytes in the mesenteric lymph node (Ohya et al., 2014), which is a driving force of the enhanced production of inflammatory cytokines, such as IFNγ. The upregulation of KCa3.1 expression is caused by HDAC2- and HDAC3-mediated epigenetic modifications as a pan-HDAC inhibitor vorinostat and selective inhibitors of HDAC2 and HDAC3 suppress the increase in transcriptional expression of KCa3.1 in the splenic CD4⁺ T cells in the DSS colitis model (Matsui et al., 2018; Fig. 1). Moreover, KCa3.1 channel blocker TRAM-34 reduces the severity of DSS-induced colitis and normalizes the expression of KCa3.1, NDPK-B, and Th1 cytokines in the mesenteric lymph node CD4^+^ T cells (Ohya et al., 2014). Similar to TRAM-34, another KCa3.1 blocker NS6180 also suppresses colon inflammation and improved body weight loss in the TNBS colitis model (Strobaek et al., 2013). Moreover, KCa3.1 KO mice also display impaired Ca^2+^ influx and cytokine production in CD4^+^ Th1 and Th2 cells, whereas T-regulatory and Th17 cell functions are not affected. Consistent with pharmacological inhibition studies, KCa3.1 KO mice are protected against developing severe colitis in mouse models of colitis induced by either T-cell transfer or TNBS (Di et al., 2010), further supporting a causative role of KCa3.1 in intestinal inflammation.

Besides KCa3.1, voltage-gated Kv1.3 channel is also expressed by human T cells and controls T-cell activation, proliferation, and cytokine production by maintaining the driving force for Ca^2+^ influx through CRAC channels. In fact, both KCa3.1 and Kv1.3 cooperatively and compensatorily regulate antigen-specific memory T cell functions (Chiang et al., 2017). In mucosal biopsies from patients with active UC, the expression level of Kv1.3 in both infiltrated CD4^+^ and CD8^+^ T cells is markedly increased, which is associated with the production of pro-inflammatory IL-17A and TNF-α (Koch Hansen et al., 2014). As Kv1.3 loss-of-function in mice is lethal, functional studies are mainly dependent on pharmacological tools. Inhibition of Kv1.3 expressed by CD4^+^ T cells with a small molecule inhibitor DES1 suppresses intestinal inflammation in a humanized mouse model of UC (Unterweger et al., 2021). Another Kv1.3 blocker PAP-1 also protects animals against DSS-induced colitis possibly through inhibition of NLRP3 inflammasome pathway downstream of Kv1.3 signaling (Mei et al., 2019). It should be noted that the specificity of these inhibitors should be carefully evaluated in the preclinical studies. Moreover, cell specific ablation of distinct K^+^ channels is also needed for mechanistic studies regarding functions of KCa3.1 and Kv1.3 in distinct cell types.

## Ion channels in intestinal immune-epithelial crosstalk

Intestine immune cells are embedded in different layers of gut lumen surrounded by numerous resident non-immune cells such as IECs, chemosensory tuft cells, and mucin-secreting goblet cells. Immune-epithelial crosstalk at the intestinal barrier surface is a central mechanism of host defense. Ion channels are broadly expressed by diverse resident non-immune cells in the GI tract which play a critical role in gut homeostasis and intestinal inflammation.

### CRAC

IEC-derived antimicrobial peptides are critical components of intestinal innate immunity (Muniz et al., 2012). A recent study demonstrated that Orai1 expressed by the pancreatic acini plays an essential role in the control of intestinal microbiota and immunity through releasing antimicrobial peptides (Ahuja et al., 2017; Tilg and Adolph, 2017). Conditional KO of Orai1 from pancreatic acini of adult mice impairs Ca^2+^ signaling and reduces the total level of cathelicidin-related peptide CRAMP. This reduction in secretion of antimicrobial peptides from pancreas causes intestinal bacterial outgrowth and dysbiosis, ultimately leading to systemic bacterial translocation, inflammation, and death, representing a novel mode of cross organ immune regulation. These dire consequences can be rescued by purified liquid diet and broad-spectrum antibiotics treatments which inhibit bacterial outgrowth and supplement of synthetic CRAMP (Ahuja et al., 2017; Tilg and Adolph, 2017).

Interestingly, the CRAC channel inhibitor BTP2 or CM4620 was shown to significantly reduce the production of inflammatory cytokines (such as IFNγ, TNF, IL-17A, IL-13, and IL-4) from human intestinal T cells, B cells, ILCs and myeloid cells, and improve colon inflammation in a mouse model of IBD (Letizia et al., 2022). Note that CM4620 is in an ongoing phase I/II clinical trial for children and young adults with acute pancreatitis (Letizia et al., 2022). Although pharmacological inhibition of SOCE does not affect the functions of human or mouse IECs in colonic organoid cultures, epithelium-expressed STIM1 regulates intestinal homeostasis through promoting the loss of goblet cells in IBD patients (Liang et al., 2022). Taken together, these studies implicated that CRAC expressed by either epithelia or immune cell may serve as an important regulator of intestinal immune function and could be potential drug targets for the treatment of IBD.

### TRP channels

TRPM5 is a cation channel mediating sweet, umami, and bitter tastes upon activation by intracellular Ca^2+^ resulting from activation of upstream G-protein-coupled taste receptors in taste buds (Dutta Banik et al., 2018). Like the taste bud cells, enteric tuft cells are chemosensory IECs expressing TRPM5 and succinate receptor 1 (SUCNR1), a GPCR activated by a microbiota-derived metabolite succinate. Upon infections, tuft cells sense succinate secreted from pathogens such as protists tritrichomonas, bacteria, and helminths, through SUCNR1 activation resulting in an increase in intracellular Ca^2+^ that subsequently activates TRPM5. Both in vivo and in vitro data showed that TRPM5 activation is critical to modulating Na^+^ influx and membrane depolarization in the tuft cells, driving the release of IL-25 that activates IL-25 receptor expressed by lamina propria ILC2s which subsequently promotes IL-13 production and release, and further induces tuft cell expansion. These findings place TRPM5 at the center of tuft cell-ILC2 signaling circuit that mediates epithelia immune interactions to fight intestinal parasite infections (Howitt et al., 2016; Nadjsombati et al., 2018; Schneider et al., 2018; O’Leary et al., 2019; Fig. 1).

TRPV4 is a non-selective cation channel-sensing cell volume dynamics and many inflammatory mediators (Toft-Bertelsen and MacAulay, 2021). Besides dominant expression in the skin and kidney, TRPV4 is also expressed by IECs and immune cells, especially macrophages (Luo et al., 2018; Rizopoulos et al., 2018). TRPV4 mRNA transcripts are significantly increased in colon biopsies from both CD and UC patients when compared with healthy subjects (Rizopoulos et al., 2018). TRPV4 mRNA expression is also significantly increased in the colon of TNBS-treated mice compared with control animals. Administrations of a selective TRPV4 antagonist RN1734 suppresses the severity of TNBS-induced colitis and visceral pain (Fichna et al., 2012). Another study showed increased TRPV4 expression in human colon samples, human IEC cell lines (Caco-2 and T84), and inflamed colons of mice induced by intracolonic administration of a TRPV4 activator 4α-phorbol-12,13-didecanoate (4αPDD), further supporting a role of TRPV4 in intestinal inflammation (D’Aldebert et al., 2011; Fig. 1). However, these pharmacological manipulations lack specificity and have potential off-target actions. Other studies using both pharmacological and genetic approaches showed that TRPV4 is upregulated in vascular endothelial cells and contributes to increased vascular permeability promoting DSS-induced colon inflammation (Matsumoto et al., 2018). Further studies showed that TRPV4 expressed by vascular endothelial cells and potentially macrophages facilitates the progression of colitis-associated cancer induced by azoxymethane/DSS (Matsumoto et al., 2020). It should be noted that TRPV4 is also expressed by muscularis macrophages in the gut (Luo et al., 2018) which are shown to be required for maintain normal GI motility. Activation of TRPV4 promotes GI motility through direct interaction between the muscularis macrophages with intestinal smooth muscles involving macrophage-derived prostaglandin E2. Moreover, genetic or pharmacological inhibition of TRPV4 can improve chemotherapy-induced GI hypermotility in mice (Luo et al., 2018). Therefore, immune cell-expressed ion channels likely have a broader function in the GI tract beyond gut immunity.

### P2X7 receptor

Human fetal small intestinal IECs express P2X7R that is activated during *Toxoplasma gondii* infection (Quan et al., 2018), which promotes the production and release of IL-1β through NLRP3 inflammasome activation. In vitro siRNA knockdown of P2X7R reduces *T. gondii*-induced IL-1β secretion and substantially increased parasite proliferation in *T. gondii*-infected FHs 74 Int epithelial cells, which is correlated with a reduction of the NLRP3 inflammasome activation, providing mechanistic insights into mucosal immune mechanisms of *T. gondii* infection (Quan et al., 2018). Interestingly, another study reported that in vitro and in vivo infections of IECs by *T. gondii* and *T. spiralis* do not promote inflammasome-associated IL-1β secretion. Instead, these parasites cause a P2X7R-dependent production of CCL5, TNF-α, and IL-6 from IECs, which recruits CD11C^+^/CD103^+^ DCs into the epithelial layer (Huang et al., 2017). Although these results suggest that P2X7R might engage multiple signaling pathways in the intestinal epithelium to mount innate immune response upon parasite infections (Fig. 1), more selective genetic and pharmacological tools are needed to validate these findings.

### K^+^ channels

Many types of K^+^ channels are expressed in the intestinal epithelium and participate in maintaining normal intestinal secretion and barrier integrity (Cosme et al., 2021). For instance, deficiency of Trek1 (K2P2.1), a two-pore-domain background potassium channel, causes barrier dysfunction of the human colon epithelial cell line T84 monolayer. Barrier dysfunction in a mouse model of ovalbumin-induced intestinal allergy is also found to be related to reduced Trek1 expression caused by activation of the MAPK pathways and HDAC1, which is rescued by HDAC1 inhibitors (Huang et al., 2016; Fig. 1).

GI inflammation also involves K^+^ channels. For instance, the K (ATP) channel Kir6.1, SUR2B is upregulated in the mesenteric lymphatic vessels in a mouse model of TNBS-induced ileitis, and the K (ATP) channel blocker glibenclamide improves lymphatic pumping through attenuating TNBS-induced membrane hyperpolarization in the mesenteric lymphatic vessels, suggesting the involvement of K (ATP) channels in TNBS-induced lymphatic contractile dysfunction (Mathias and von der Weid, 2013). Moreover, LRRC26, a regulatory subunit of the Ca^2+^- and voltage-activated BK channel, is functionally expressed by the mucin-secreting goblet cells in the GI tract and LRRC26-associated BK channels contribute to the resting transepithelial current across mouse distal colonic mucosa. LRRC26 deficiency renders inactivity of normally expressed BK channels at physiological conditions. Deficiency in LRRC26 or BK pore-forming α-subunit dramatically exacerbates DSS-induced colitis, suggesting a protective role of LRRC26-associated BK channels against chemical-induced colitis in mice, potentially through maintaining normal function of the goblet cells and mucin secretion (Gonzalez-Perez et al., 2021; Fig. 1).

## Ion channels in intestinal neuroimmune interactions

The GI tract is innervated by both extrinsic and intrinsic nervous systems (Phillips and Powley, 2007). ANS receives signals from the CNS to regulate GI function while sensory neurons from dorsal root and vagal ganglia detect danger signals in the gut lumen to initiate protective responses. Moreover, ENS, the second brain positioned along the gut wall, comprises a full repertoire of sensory neurons, interneurons, motor neurons, and glial cells which collectively detect luminal contents, drive secretory function and intestinal motility, and maintain immune homeostasis and GI barrier integrity (Adamantidis et al., 2014; Camilleri, 2021; Niesler et al., 2021; Wang et al., 2022). These distinct neuronal components and their signal transducers participate in regulating GI inflammation and host defense (Fig. 2).

### Vagal-immune interactions

Sympathetic input suppresses colon motility by acting on intrinsic myenteric neurons while regulates gut immune function through releasing noradrenaline that acts on G-protein-coupled α and β adrenergic receptors expressed by enteric neurons and immune cells (Populin et al., 2021; Smith-Edwards et al., 2021). The vagus nerve, on the other hand, modulates intestinal inflammation through the key mediator of the cholinergic anti-inflammatory pathway, acetylcholine (ACh), acting primarily on nAChRs and driving Na^+^ influx and membrane depolarization (Van Der Zanden et al., 2009a), regulating cytokine release from immune cells (Rueda Ruzafa et al., 2021). Most of the vagus nerve-mediated anti-inflammatory responses in the GI tract are mediated by α7nAChR (Cailotto et al., 2014; Ji et al., 2014; Matteoli et al., 2014; Rana et al., 2018; Bosmans et al., 2019; Yang et al., 2021), although α4/β2 nAChRs are also reported to promote macrophage phagocytosis in vitro and potentially increases uptake of luminal bacteria by lamina propria macrophages in response to vagus nerve stimulation (VNS) in mice (van der Zanden et al., 2009b; Fig. 2).

In mouse models of IBD, VNS modulates intestinal inflammation through a centrally mediated vagal modulation mechanism (Cheng et al., 2020). Besides VNS, chemical stimulation of the central cholinergic pathway has also been used to drive ACh–nAChR signaling in the GI tract (Ji et al., 2014) to reduce mucosal inflammation in mouse models of colitis induced by DSS or TNBS. This modulatory effect is abolished in mice with vagotomy, splenic neurectomy, or splenectomy, suggesting the involvement of a vagus nerve-to-spleen circuit and α7nAChR signaling pathway (Ji et al., 2014). Innate immune cells such as resident macrophages and mast cells are critical targets for the vagus nerve-driving anti-inflammatory response in the GI tract, for instance, VNS dampens intestinal inflammation in a murine model of experimental FA in a α7nAChR-independent manner, possibly through dampening mast cell activity and increasing phagocytosis of ovalbumin by CX3CR1^+^ macrophages (Bosmans et al., 2019). In a mouse model of postoperative ileus (POI), VNS driven by electroacupuncture causes the release of ACh that activates the α7nAChR-mediated JAK2/STAT3 signaling pathway in macrophages, suppressing the production of inflammatory cytokines (Yang et al., 2021). VNS attenuates surgery-induced intestinal inflammation and improves postoperative intestinal transit in wild-type, splenic denervated, and T-cell-deficient mice, suggesting that the vagal nerve-mediated anti-inflammatory effect in the intestine is likely independent of the spleen and T cells (Matteoli et al., 2014).

### Nociceptor-immune interactions

Visceral sensory neurons residing in the DRG that have sensory nerve endings project to the gut wall and mesentery mediating mechanosensory and chemosensory responses in the GI tract (Spencer and Hu, 2020). Accumulating evidence indicates that there is a bi-directional communication between these sensory nerve endings and immune cells, driving aberrant neuroimmune interactions which can lead to visceral hypersensitivity and GI inflammation (Boeckxstaens and Wouters, 2017). This neuro-immune regulation is largely orchestrated through nociceptor-expressed TRP channels and nociceptor-derived neuropeptides (Lai et al., 2017).

In DSS-induced colitis model, the TRPV1/CGRP-positive nerve fiber density increases in the distal colon wall and TRPV1 activation-induced release of CGRP/SP from the distal colon is greater than that from the proximal colon, which is further enhanced in the setting of colitis. TRPV1 expression is increased in DRGs projecting to the distal colon when compared to that in the proximal colon, which is also further enhanced during colitis (Engel et al., 2012). This expression pattern is correlated with the phenomenon showing the proximodistal aggravation of inflammation in the DSS colitis model. Consistent with the importance of TRP channels and neuropeptides in GI inflammation, SP deficiency and RTX-mediated ablation of the TRPV1^+^ fibers improve DSS colitis without the presence of the proximodistal gradient of inflammation, suggesting substance P released from the TRPV1-expressing sensory neurons is required for this phenomenon (Engel et al., 2012). Genetic ablation of TRPV1 or TRPA1 significantly attenuates the severity of colitis (Szitter et al., 2010; Utsumi et al., 2018). Moreover, DSS treatment up-regulates both CGRP^+^ and SP^+^ sensory nerve fibers and TRPA1 or TRPV1 KO mice have less upregulation of SP^+^ but not the CGRP^+^ sensory nerve fibers, suggesting that DSS-induced colitis is driven by TRPA1- and TRPV1-mediated release of SP (Utsumi et al., 2018; Fig. 2). On the other hand, another study reported that capsaicin-induced denervation of the TRPV1^+^ extrinsic sensory fibers in neonatal mice exacerbates oxazolone-induced colitis without significant changes in the expression of CGRP^+^ or SP^+^ nerve fibers in the colon, suggesting a dispensable role of neuropeptides and the involvement of additional nociceptor-derived neurotransmitters in the pathogenesis of the colitis (Lee et al., 2012).

TRPA1 is reported to be a major mediator of the TNBS-induced colitis (Engel et al., 2011). Mechanistically, TNBS directly activates TRPA1 through covalent modification of cysteine and lysine residues in the cytoplasmic N-terminus of the channel. Application of TNBS activates inward currents, promotes Ca^2+^ influx and release of SP from cultured DRG neurons in a TRPA1-dependent manner. Importantly, genetic or pharmacological ablation of TRPA1 function suppresses both TNBS- and DSS-induce colitis, supporting a critical role of TRPA1-mediated SP release in the pathogenesis of colitis (Engel et al., 2011; Fig. 2).

In addition to chemical-induced colitis, TRPV1^+^ neurons also protect against infection caused by an enteric bacterial pathogen *Citrobacter rodentium* as RTX-induced ablation of the TRPV1^+^ fibers increases fecal and colonic adherent *C. rodentium* on day 10 post-infection (p.i.) until day 29 p.i., which is recapitulated by genetic ablation of the TRPV1 channel function (Ramirez et al., 2020). It is proposed that bacteria-mediated activation of TLR4 receptor sensitizes TRPV1 channels and subsequently causes release of CGRP which plays an anti-inflammatory role in maintaining mucosal homeostasis in the GI tract (Assas et al., 2014; Fig. 2).

Besides TRPA1/TRPV1, TRPM8 is also functionally expressed by the colon-innervating DRG neurons and TRPM8 expression is upregulated in inflamed colon samples from both human and mouse models of DSS- and TNBS-induced colitis (Harrington et al., 2011). TRPM8 KO mice display enhanced levels of inflammatory neuropeptides in the colon although the inflammation is not significantly altered when compared with wild-type mice. Systemic activation of TRPM8 by icilin suppresses intestinal inflammation in wild-type mice but not in TRPM8 KO mice in chemically induced colitis models. Icilin also reduces the levels of inflammatory cytokines in the colon of TNBS-treated mice. The level of CGRP in the colon of DSS-treated TRPM8 KO mice is increased when compared with that in the wild-type mice. Interestingly, application of icilin can block capsaicin-induced CGRP release in ex vivo colon tissues. These findings suggest that TRPM8 activation suppresses CGRP release from the TRPV1^+^ nociceptors to execute an anti-inflammatory response (Ramachandran et al., 2013; Fig. 2). However, it is not understood why icilin activation of TRPM8 does not promote CGRP release since published studies showed that activation of TRPM8 is correlated with CGRP release from DRG neurons and TRPM8 protects against intestinal injury and mucosal inflammation through CGRP (de Jong et al., 2015; Fouad et al., 2021). It is also important to distinguish the function of TRP channels in immune cells from primary nociceptors in the setting of intestinal inflammation as both cell types are critically involved in the pathogenesis of chemical-induced GI inflammation. Selective ablation of ion channel function in distinct nociceptors and immune components using conditional KO approaches can be used to address this issue.

### ENS-immune interactions

Emerging evidence suggests that enteric neurons expressing choline acetyltransferase (ChAT) play a critical role in gut immunity and inflammation as evidenced by a reduction of both cholinergic neurons and ACh in IBDs (Zheng et al., 2021). Direct activation of the ChAT^+^ enteric neurons triggers the release of ACh that subsequently acts on the α7nAChR expressed by monocytes/macrophages to inhibit intestinal inflammation (Tsuchida et al., 2011). Mechanistically, ACh activates nAChRs on monocytic myeloid-derived suppressor cells to promote the release of anti-inflammatory cytokine IL-10 through the ERK signaling (Zheng et al., 2021). The involvement of cholinergic enteric neuron-α7nAChR-expressing macrophage signaling axis has been proposed to mediate the beneficial effects of 5-hydroxytryptamine receptor 4 (5-HT₄R) agonists such as mosapride citrate (MOS), CJ-033466, and prucalopride on GI inflammation, especially after abdominal surgery in a mouse model of POI (Tsuchida et al., 2011; Stakenborg et al., 2019). This action of 5-HT₄R agonists can be recapitulated by the inhibitory effect of electrical field stimulation (EFS) on macrophage function, which is attenuated by the neurotransmission blocker tetrodotoxin (TTX) and the N-type VGCC inhibitor ω-conotoxin (Stakenborg et al., 2019; Fig. 2). However, if selective activation of 5-HT₄R-expressing cholinergic enteric neurons in vivo by 5-HT₄R agonists or EFS is sufficient to inhibit POI is not known as these manipulations are not specific considering the widespread expression of 5-HT₄R and non-specific activation of diverse types of enteric neurons by EFS. Future studies using neurogenetic techniques to gain genetic access to distinct enteric neuron populations for both gain- and loss-of-function manipulations are required to validate these findings.

More interestingly, recent studies have identified an indirect vagal nerve-cholinergic myenteric neurons–muscularis macrophages axis in the modulation of GI inflammation. Histological studies showed that vagal nerve endings surround enteric neurons expressing nNOS, vasoactive intestinal peptide, and ChAT (Cailotto et al., 2014). Moreover, vagus nerve interacts with cholinergic myenteric neurons which are in close contact with the α7nAChR-expressing muscularis macrophages (Matteoli et al., 2014; Fig. 2). In a mouse model of FA produced by repeated oral ovalbumin administration, VNS and cholinergic activation with α7nAChR agonist GTS-21, or nicotine can suppress the allergic symptoms through dampening mast cell hyperplasia and expression of Th1 and Th2 cytokines in the colon. These beneficial effects are not mediated by systemic immune modulation, further supporting the involvement of a localized α7nAChR-mediated cholinergic neuroimmune interaction between VNS, ENS activation, and mucosal mast cell function (Matteoli et al., 2014). On the other hand, these histological studies are indirect evidence for potential interactions between vagal and enteric neurons in the gut. Direct evidence of functional interactions based on electrophysiological recordings and neurotransmitter release are needed to support these conclusions.

## Conclusions

In the past decades, significant progresses have been made in understanding the cells, molecules, and cell–cell interactions in gut immunity. It is clear that ion channels play an essential role in maintaining gut immune homeostasis under physiological conditions as well as inflammatory GI disorders including IBD. Although expression and function of some specific ion channels such as TRP channels in intestinal immune cells need to be re-evaluated, convincing evidence has been provided to demonstrate that many ion channels, especially Orai channels, Kv1.3, P2X7R, and nAChR, are extensively expressed by CD4^+^, CD8^+^, and Tregs and play critical roles in expansion and differentiation of distinct immune cell subsets as well as immune tolerance at the steady state in gut immunity. Dysregulation of these ion channels in distinct immune cell subsets also critically contribute to intestinal inflammatory disorders.

However, there are two important questions that need to be answered by future studies. First, can we use ion channel expression profiles as biomarkers for intestinal inflammatory disorders, such as IBD? Ion channels could be a good candidate to assess the risk and prognosis and evaluate treatment options for inflammatory diseases in the GI tract based on their expression patterns and functions in gut immunity. The use of ion channel marker-assisted diagnosis and targeted therapies derived from an individual’s molecular profile of ion channels should facilitate the application of personalized medicine. Therefore, it will be important to perform a big data-based comparison of ion channel properties between different conditions (male versus female, aging versus adult, physiology versus pathology) to fine-tune the correlation between ion channel expression and function with GI inflammatory disorders.

Secondly, can the basic ion channel mechanisms in gut immunity unveiled in animal studies be translated into clinical trials using specific ion channel blockers? In general, the translation of findings from animal models to clinical practice has not been very successful. Therefore, experimental designs of preclinical animal studies need to be improved and humanized animal models of GI disorders might enhance translational potentials (Unterweger et al., 2021). This is especially exciting because ongoing high throughput drug screening has identified numerous activators and inhibitors that have the potential to be used in the treatment of GI inflammatory disorders by targeting ion channels such as Orai channels, Kv1.3, P2X7R, and nAChR, which are the most important channels in gut immunity that have emerged over the past decades. Among them, Orai channels, Kv1.3 are the most promising ion channel drug targets for the treatment of IBD as shown by recent preclinical studies (Unterweger et al., 2021; Letizia et al., 2022). On the other hand, it remains a big challenge to target distinct cell types when the same ion channels are expressed by various tissues in the gut to avoid any off-target side effects and toxicity issues associated with ion channel activators and blockers. Alternatively, VNS produced by electrical stimulation and acupuncture points is a promising alternative treatment approach for IBD through nAChR-mediated cholinergic anti-inflammatory pathways (de Araujo and de Lartigue, 2020; Lei and Duan, 2021).

## References

[bib1] Adamantidis, A.R., F. Zhang, L. de Lecea, and K. Deisseroth. 2014. Optogenetics: Opsins and optical interfaces in neuroscience. Cold Spring Harb. Protoc. 2014:815–822. 10.1101/pdb.top083329 25086025

[bib2] Ahuja, M., D.M. Schwartz, M. Tandon, A. Son, M. Zeng, W. Swaim, M. Eckhaus, V. Hoffman, Y. Cui, B. Xiao, . 2017. Orai1-Mediated antimicrobial secretion from pancreatic acini shapes the gut microbiome and regulates gut innate immunity. Cell Metabol. 25:635–646. 10.1016/j.cmet.2017.02.007 PMC5345693 28273482

[bib3] Alarcon-Vila, C., A. Baroja-Mazo, C. de Torre-Minguela, C.M. Martinez, J.J. Martinez-Garcia, H. Martinez-Banaclocha, C. Garcia-Palenciano, and P. Pelegrin. 2020. CD14 release induced by P2X7 receptor restricts inflammation and increases survival during sepsis. Elife. 9:e60849. 10.7554/eLife.60849 33135636 PMC7690950

[bib4] Antonini, M., M. Lo Conte, C. Sorini, and M. Falcone. 2019. How the interplay between the commensal microbiota, gut barrier integrity, and mucosal immunity regulates brain autoimmunity. Front. Immunol. 10:1937. 10.3389/fimmu.2019.01937 31475000 PMC6706873

[bib5] Assas, B.M., J.A. Miyan, and J.L. Pennock. 2014. Cross-talk between neural and immune receptors provides a potential mechanism of homeostatic regulation in the gut mucosa. Mucosal Immunol. 7:1283–1289. 10.1038/mi.2014.80 25183366

[bib6] Bai, L., S. Mesgarzadeh, K.S. Ramesh, E.L. Huey, Y. Liu, L.A. Gray, T.J. Aitken, Y. Chen, L.R. Beutler, J.S. Ahn, . 2019. Genetic identification of vagal sensory neurons that control feeding. Cell. 179:1129–1143.e23. 10.1016/j.cell.2019.10.031 31730854 PMC6916730

[bib7] Beceiro, S., J.N. Radin, R. Chatuvedi, M.B. Piazuelo, D.J. Horvarth, H. Cortado, Y. Gu, B. Dixon, C. Gu, I. Lange, . 2017. TRPM2 ion channels regulate macrophage polarization and gastric inflammation during *Helicobacter pylori* infection. Mucosal Immunol. 10:493–507. 10.1038/mi.2016.60 27435104 PMC5250617

[bib8] Beesetty, P., K.B. Wieczerzak, J.N. Gibson, T. Kaitsuka, C.T. Luu, M. Matsushita, and J.A. Kozak. 2018. Inactivation of TRPM7 kinase in mice results in enlarged spleens, reduced T-cell proliferation and diminished store-operated calcium entry. Sci. Rep. 8:3023. 10.1038/s41598-018-21004-w 29445164 PMC5813043

[bib9] Bertin, S., Y. Aoki-Nonaka, P.R. de Jong, L.L. Nohara, H. Xu, S.R. Stanwood, S. Srikanth, J. Lee, K. To, L. Abramson, . 2014. The ion channel TRPV1 regulates the activation and proinflammatory properties of CD4^+^ T cells. Nat. Immunol. 15:1055–1063. 10.1038/ni.3009 25282159 PMC4843825

[bib10] Bertin, S., Y. Aoki-Nonaka, J. Lee, P.R. de Jong, P. Kim, T. Han, T. Yu, K. To, N. Takahashi, B.S. Boland, . 2017. The TRPA1 ion channel is expressed in CD4^+^ T cells and restrains T-cell-mediated colitis through inhibition of TRPV1. Gut. 66:1584–1596. 10.1136/gutjnl-2015-310710 27325418 PMC5173457

[bib11] Beyder, A., and G. Farrugia. 2016. Ion channelopathies in functional GI disorders. Am. J. Physiol. Gastrointest. Liver Physiol. 311:G581–G586. 10.1152/ajpgi.00237.2016 27514480 PMC5142191

[bib12] Bhattacharya, A., Q. Wang, H. Ao, J.R. Shoblock, B. Lord, L. Aluisio, I. Fraser, D. Nepomuceno, R.A. Neff, N. Welty, . 2013. Pharmacological characterization of a novel centrally permeable P2X7 receptor antagonist: JNJ-47965567. Br. J. Pharmacol. 170:624–640. 10.1111/bph.12314 23889535 PMC3792000

[bib13] Boeckxstaens, G.E., and M.M. Wouters. 2017. Neuroimmune factors in functional gastrointestinal disorders: A focus on irritable bowel syndrome. Neurogastroenterol. Motil. 29:e13007. 10.1111/nmo.13007 28027594

[bib14] Bosmans, G., I. Appeltans, N. Stakenborg, P.J. Gomez-Pinilla, M.V. Florens, J. Aguilera-Lizarraga, G. Matteoli, and G.E. Boeckxstaens. 2019. Vagus nerve stimulation dampens intestinal inflammation in a murine model of experimental food allergy. Allergy. 74:1748–1759. 10.1111/all.13790 30897213 PMC6790670

[bib15] Braga Ferreira, L.G., J.V. Faria, J.P.S. Dos Santos, and R.X. Faria. 2020. Capsaicin: TRPV1-independent mechanisms and novel therapeutic possibilities. Eur. J. Pharmacol. 887:173356. 10.1016/j.ejphar.2020.173356 32763303

[bib16] Browning, K.N., and R.A. Travagli. 2014. Central nervous system control of gastrointestinal motility and secretion and modulation of gastrointestinal functions. Compr. Physiol. 4:1339–1368. 10.1002/cphy.c130055 25428846 PMC4858318

[bib17] Cahalan, M.D., and K.G. Chandy. 2009. The functional network of ion channels in T lymphocytes. Immunol. Rev. 231:59–87. 10.1111/j.1600-065X.2009.00816.x 19754890 PMC3133616

[bib18] Cailotto, C., P.J. Gomez-Pinilla, L.M. Costes, J. van der Vliet, M. Di Giovangiulio, A. Nemethova, G. Matteoli, and G.E. Boeckxstaens. 2014. Neuro-anatomical evidence indicating indirect modulation of macrophages by vagal efferents in the intestine but not in the spleen. PLoS One. 9:e87785. 10.1371/journal.pone.0087785 24489965 PMC3906221

[bib19] Camilleri, M. 2021. Gastrointestinal motility disorders in neurologic disease. J. Clin. Invest. 131:e143771. 10.1172/JCI143771 33586685 PMC7880310

[bib20] Chassaing, B., M. Kumar, M.T. Baker, V. Singh, and M. Vijay-Kumar. 2014. Mammalian gut immunity. Biomed. J. 37:246–258. 10.4103/2319-4170.130922 25163502 PMC4714863

[bib21] Chen, W., D. Liu, C. Ren, X. Su, C.K. Wong, and R. Yang. 2022. A special network comprised of macrophages, epithelial cells, and gut microbiota for gut homeostasis. Cells. 11:307. 10.3390/cells11020307 35053422 PMC8774616

[bib22] Cheng, J., H. Shen, R. Chowdhury, T. Abdi, F. Selaru, and J.D.Z. Chen. 2020. Potential of electrical neuromodulation for inflammatory bowel disease. Inflamm. Bowel Dis. 26:1119–1130. 10.1093/ibd/izz289 31782957

[bib23] Cheng, N., L. Zhang, and L. Liu. 2021. Understanding the role of purinergic P2X7 receptors in the gastrointestinal system: A systematic review. Front. Pharmacol. 12:786579. 10.3389/fphar.2021.786579 34987401 PMC8721002

[bib24] Chiang, E.Y., T. Li, S. Jeet, I. Peng, J. Zhang, W.P. Lee, J. DeVoss, P. Caplazi, J. Chen, S. Warming, . 2017. Potassium channels Kv1.3 and KCa3.1 cooperatively and compensatorily regulate antigen-specific memory T cell functions. Nat. Commun. 8:14644. 10.1038/ncomms14644 28248292 PMC5337993

[bib25] Cosme, D., M.M. Estevinho, F. Rieder, and F. Magro. 2021. Potassium channels in intestinal epithelial cells and their pharmacological modulation: A systematic review. Am. J. Physiol. Cell Physiol. 320:C520–C546. 10.1152/ajpcell.00393.2020 33326312 PMC8424539

[bib26] D’Aldebert, E., N. Cenac, P. Rousset, L. Martin, C. Rolland, K. Chapman, J. Selves, L. Alric, J.P. Vinel, and N. Vergnolle. 2011. Transient receptor potential vanilloid 4 activated inflammatory signals by intestinal epithelial cells and colitis in mice. Gastroenterology. 140:275–285. 10.1053/j.gastro.2010.09.045 20888819

[bib27] Dayam, R.M., A. Saric, R.E. Shilliday, and R.J. Botelho. 2015. The phosphoinositide-gated lysosomal Ca^2+^ channel, TRPML1, is required for phagosome maturation. Traffic. 16:1010–1026. 10.1111/tra.12303 26010303

[bib28] Dayam, R.M., C.X. Sun, C.H. Choy, G. Mancuso, M. Glogauer, and R.J. Botelho. 2017. The lipid kinase PIKfyve coordinates the neutrophil immune response through the activation of the rac GTPase. J. Immunol. 199:2096–2105. 10.4049/jimmunol.1601466 28779020

[bib29] de Araujo, A., and G. de Lartigue. 2020. Non-canonical cholinergic anti-inflammatory pathway in IBD. Nat. Rev. Gastroenterol. Hepatol. 17:651–652. 10.1038/s41575-020-0356-y 32759984 PMC7826200

[bib30] de Campos, N.E., C. Marques-da-Silva, G. Correa, M.T. Castelo-Branco, H.S. de Souza, and R. Coutinho-Silva. 2012. Characterizing the presence and sensitivity of the P2X7 receptor in different compartments of the gut. J. Innate Immun. 4:529–541. 10.1159/000336628 22508425 PMC6741528

[bib31] de Jong, P.R., N. Takahashi, M. Peiris, S. Bertin, J. Lee, M.G. Gareau, A. Paniagua, A.R. Harris, D.S. Herdman, M. Corr, . 2015. TRPM8 on mucosal sensory nerves regulates colitogenic responses by innate immune cells via CGRP. Mucosal Immunol. 8:491–504. 10.1038/mi.2014.82 25269705 PMC4382463

[bib32] Di, L., S. Srivastava, O. Zhdanova, Y. Ding, Z. Li, H. Wulff, M. Lafaille, and E.Y. Skolnik. 2010. Inhibition of the K^+^ channel KCa3.1 ameliorates T cell-mediated colitis. Proc. Natl. Acad. Sci. USA. 107:1541–1546. 10.1073/pnas.0910133107 20080610 PMC2824388

[bib33] Di Sabatino, A., L. Rovedatti, R. Kaur, J.P. Spencer, J.T. Brown, V.D. Morisset, P. Biancheri, N.A. Leakey, J.I. Wilde, L. Scott, . 2009. Targeting gut T cell Ca^2+^ release-activated Ca^2+^ channels inhibits T cell cytokine production and T-box transcription factor T-bet in inflammatory bowel disease. J. Immunol. 183:3454–3462. 10.4049/jimmunol.0802887 19648266

[bib34] Duo, L., T. Wu, Z. Ke, L. Hu, C. Wang, G. Teng, W. Zhang, W. Wang, Q. Ge, Y. Yang, and Y. Dai. 2020. Gain of function of ion channel TRPV1 exacerbates experimental colitis by promoting dendritic cell activation. Mol. Ther. Nucleic Acids. 22:924–936. 10.1016/j.omtn.2020.10.006 33251043 PMC7666365

[bib35] Dutta Banik, D., L.E. Martin, M. Freichel, A.M. Torregrossa, and K.F. Medler. 2018. TRPM4 and TRPM5 are both required for normal signaling in taste receptor cells. Proc. Natl. Acad. Sci. USA. 115:E772–E781. 10.1073/pnas.1718802115 29311301 PMC5789955

[bib36] Engel, M.A., M. Khalil, S.M. Mueller-Tribbensee, C. Becker, W.L. Neuhuber, M.F. Neurath, and P.W. Reeh. 2012. The proximodistal aggravation of colitis depends on substance P released from TRPV1-expressing sensory neurons. J. Gastroenterol. 47:256–265. 10.1007/s00535-011-0495-6 22080974

[bib37] Engel, M.A., A. Leffler, F. Niedermirtl, A. Babes, K. Zimmermann, M.R. Filipović, I. Izydorczyk, M. Eberhardt, T.I. Kichko, S.M. Mueller-Tribbensee, . 2011. TRPA1 and substance P mediate colitis in mice. Gastroenterology. 141:1346–1358. 10.1053/j.gastro.2011.07.002 21763243

[bib38] Eser, A., J.F. Colombel, P. Rutgeerts, S. Vermeire, H. Vogelsang, M. Braddock, T. Persson, and W. Reinisch. 2015. Safety and efficacy of an oral inhibitor of the purinergic receptor P2X7 in adult patients with moderately to severely active Crohn’s disease: A randomized placebo-controlled, double-blind, phase IIa study. Inflamm. Bowel Dis. 21:2247–2253. 10.1097/MIB.0000000000000514 26197451

[bib39] Feske, S., H. Wulff, and E.Y. Skolnik. 2015. Ion channels in innate and adaptive immunity. Annu. Rev. Immunol. 33:291–353. 10.1146/annurev-immunol-032414-112212 25861976 PMC4822408

[bib40] Fichna, J., A. Mokrowiecka, A.I. Cygankiewicz, P.K. Zakrzewski, E. Małecka-Panas, A. Janecka, W.M. Krajewska, and M.A. Storr. 2012. Transient receptor potential vanilloid 4 blockade protects against experimental colitis in mice: A new strategy for inflammatory bowel diseases treatment? Neurogastroenterol. Motil. 24:e557–e560. 10.1111/j.1365-2982.2012.01999.x 22882778

[bib41] Figliuolo, V.R., L.E.B. Savio, H. Safya, H. Nanini, C. Bernardazzi, A. Abalo, H.S.P. de Souza, J. Kanellopoulos, P. Bobe, C.M.L.M. Coutinho, and R. Coutinho-Silva. 2017. P2X7 receptor promotes intestinal inflammation in chemically induced colitis and triggers death of mucosal regulatory T cells. Biochim. Biophys. Acta Mol. Basis Dis. 1863:1183–1194. 10.1016/j.bbadis.2017.03.004 28286160

[bib42] Fouad, A., K. Matsumoto, K. Amagase, H. Yasuda, M. Tominaga, and S. Kato. 2021. Protective effect of TRPM8 against indomethacin-induced small intestinal injury via the release of calcitonin gene-related peptide in mice. Biol. Pharm. Bull. 44:947–957. 10.1248/bpb.b21-00045 34193690

[bib43] Froghi, S., C.R. Grant, R. Tandon, A. Quaglia, B. Davidson, and B. Fuller. 2021. New insights on the role of TRP channels in calcium signalling and immunomodulation: Review of pathways and implications for clinical practice. Clin. Rev. Allergy Immunol. 60:271–292. 10.1007/s12016-020-08824-3 33405100 PMC7985118

[bib44] Fuentes, I.M., and J.A. Christianson. 2016. Ion channels, ion channel receptors, and visceral hypersensitivity in irritable bowel syndrome. Neurogastroenterol. Motil. 28:1613–1618. 10.1111/nmo.12979 27781369 PMC5123675

[bib45] Furness, J.B. 2012. The enteric nervous system and neurogastroenterology. Nat. Rev. Gastroenterol. Hepatol. 9:286–294. 10.1038/nrgastro.2012.32 22392290

[bib46] Gees, M., B. Colsoul, and B. Nilius. 2010. The role of transient receptor potential cation channels in Ca^2+^ signaling. Cold Spring Harb. Perspect. Biol. 2:a003962. 10.1101/cshperspect.a003962 20861159 PMC2944357

[bib47] Gershon, M.D., and K.G. Margolis. 2021. The gut, its microbiome, and the brain: Connections and communications. J. Clin. Invest. 131:e143768. 10.1172/JCI143768 34523615 PMC8439601

[bib48] Giuffrida, P., and A. Di Sabatino. 2020. Targeting T cells in inflammatory bowel disease. Pharmacol. Res. 159:105040. 10.1016/j.phrs.2020.105040 32585338

[bib49] Gonzalez-Perez, V., P.L. Martinez-Espinosa, M. Sala-Rabanal, N. Bharadwaj, X.M. Xia, A.C. Chen, D. Alvarado, J.K. Gustafsson, H. Hu, M.A. Ciorba, and C.J. Lingle. 2021. Goblet cell LRRC26 regulates BK channel activation and protects against colitis in mice. Proc. Natl. Acad. Sci. USA. 118:e2019149118. 10.1073/pnas.2019149118 33431687 PMC7826367

[bib50] Gu, M., D.R. Samuelson, N.M. de la Rua, T.P. Charles, C.M. Taylor, M. Luo, R.W. Siggins, J.E. Shellito, and D.A. Welsh. 2022. Host innate and adaptive immunity shapes the gut microbiota biogeography. Microbiol. Immunol. 66:330–341. 10.1111/1348-0421.12963 35067963 PMC9189012

[bib51] Guan, F., W. Jiang, Y. Bai, X. Hou, C. Jiang, C. Zhang, M.L. Jacques, W. Liu, and J. Lei. 2021. Purinergic P2X7 receptor mediates the elimination of Trichinella spiralis by activating NF-κB/NLRP3/IL-1β pathway in macrophages. Infect. Immun. 89:e006833-20. 10.1128/IAI.00683-20 PMC8091101 33558327

[bib52] Harrington, A.M., P.A. Hughes, C.M. Martin, J. Yang, J. Castro, N.J. Isaacs, A.L. Blackshaw, and S.M. Brierley. 2011. A novel role for TRPM8 in visceral afferent function. Pain. 152:1459–1468. 10.1016/j.pain.2011.01.027 21489690

[bib53] Hinman, A., H.H. Chuang, D.M. Bautista, and D. Julius. 2006. TRP channel activation by reversible covalent modification. Proc. Natl. Acad. Sci. USA. 103:19564–19568. 10.1073/pnas.0609598103 17164327 PMC1748265

[bib54] Hofman, P., J. Cherfils-Vicini, M. Bazin, M. Ilie, T. Juhel, X. Hebuterne, E. Gilson, A. Schmid-Alliana, O. Boyer, S. Adriouch, and V. Vouret-Craviari. 2015. Genetic and pharmacological inactivation of the purinergic P2RX7 receptor dampens inflammation but increases tumor incidence in a mouse model of colitis-associated cancer. Cancer Res. 75:835–845. 10.1158/0008-5472.CAN-14-1778 25564520

[bib55] Howitt, M.R., S. Lavoie, M. Michaud, A.M. Blum, S.V. Tran, J.V. Weinstock, C.A. Gallini, K. Redding, R.F. Margolskee, L.C. Osborne, . 2016. Tuft cells, taste-chemosensory cells, orchestrate parasite type 2 immunity in the gut. Science. 351:1329–1333. 10.1126/science.aaf1648 26847546 PMC5528851

[bib56] Huang, H., J.Q. Liu, Y. Yu, L.H. Mo, R.T. Ge, H.P. Zhang, Z.G. Liu, P.Y. Zheng, and P.C. Yang. 2016. Regulation of TWIK-related potassium channel-1 (Trek1) restitutes intestinal epithelial barrier function. Cell. Mol. Immunol. 13:110–118. 10.1038/cmi.2014.137 25683610 PMC4711681

[bib57] Huang, S.W., C. Walker, J. Pennock, K. Else, W. Muller, M.J. Daniels, C. Pellegrini, D. Brough, G. Lopez-Castejon, and S.M. Cruickshank. 2017. P2X7 receptor-dependent tuning of gut epithelial responses to infection. Immunol. Cell Biol. 95:178–188. 10.1038/icb.2016.75 27559003 PMC5181772

[bib58] Huang, Y., P.A. Winkler, W. Sun, W. Lu, and J. Du. 2018. Architecture of the TRPM2 channel and its activation mechanism by ADP-ribose and calcium. Nature. 562:145–149. 10.1038/s41586-018-0558-4 30250252

[bib59] Inada, H., T. Iida, and M. Tominaga. 2006. Different expression patterns of TRP genes in murine B and T lymphocytes. Biochem. Biophys. Res. Commun. 350:762–767. 10.1016/j.bbrc.2006.09.111 17027915

[bib60] Issa, C.M., B.D. Hambly, Y. Wang, S. Maleki, W. Wang, J. Fei, and S. Bao. 2014. TRPV2 in the development of experimental colitis. Scand. J. Immunol. 80:307–312. 10.1111/sji.12206 24965783

[bib61] Ji, H., M.F. Rabbi, B. Labis, V.A. Pavlov, K.J. Tracey, and J.E. Ghia. 2014. Central cholinergic activation of a vagus nerve-to-spleen circuit alleviates experimental colitis. Mucosal Immunol. 7:335–347. 10.1038/mi.2013.52 23881354 PMC3859808

[bib62] Julius, D. 2013. TRP channels and pain. Annu. Rev. Cell Dev. Biol. 29:355–384. 10.1146/annurev-cellbio-101011-155833 24099085

[bib63] Kaymak, T., P. Hruz, and J.H. Niess. 2021. Immune system and microbiome in the esophagus: Implications for understanding inflammatory diseases. FEBS J. 289:4758–4772. 10.1111/febs.16103 34213831 PMC9542113

[bib64] Kemeny, A., X. Kodji, S. Horvath, R. Komlodi, É. Szőke, Z. Sandor, A. Perkecz, C. Gyomorei, G. Setalo, B. Kelemen, . 2018. TRPA1 acts in a protective manner in imiquimod-induced psoriasiform dermatitis in mice. J. Invest. Dermatol. 138:1774–1784. 10.1016/j.jid.2018.02.040 29550417

[bib65] Kern, B., U. Jain, C. Utsch, A. Otto, B. Busch, L. Jimenez-Soto, D. Becher, and R. Haas. 2015. Characterization of *Helicobacter pylori* VacA-containing vacuoles (VCVs), VacA intracellular trafficking and interference with calcium signalling in T lymphocytes. Cell. Microbiol. 17:1811–1832. 10.1111/cmi.12474 26078003

[bib66] Khalil, M., A. Babes, R. Lakra, S. Forsch, P.W. Reeh, S. Wirtz, C. Becker, M.F. Neurath, and M.A. Engel. 2016. Transient receptor potential melastatin 8 ion channel in macrophages modulates colitis through a balance-shift in TNF-α and interleukin-10 production. Mucosal Immunol. 9:1500–1513. 10.1038/mi.2016.16 26982596

[bib67] Kikuchi, H., J. Itoh, and S. Fukuda. 2008. Chronic nicotine stimulation modulates the immune response of mucosal T cells to Th1-dominant pattern via nAChR by upregulation of Th1-specific transcriptional factor. Neurosci. Lett. 432:217–221. 10.1016/j.neulet.2007.12.027 18248893

[bib68] Knowles, H., Y. Li, and A.L. Perraud. 2013. The TRPM2 ion channel, an oxidative stress and metabolic sensor regulating innate immunity and inflammation. Immunol. Res. 55:241–248. 10.1007/s12026-012-8373-8 22975787

[bib69] Koch Hansen, L., L. Sevelsted-Moller, M. Rabjerg, D. Larsen, T.P. Hansen, L. Klinge, H. Wulff, T. Knudsen, J. Kjeldsen, and R. Kohler. 2014. Expression of T-cell KV1.3 potassium channel correlates with pro-inflammatory cytokines and disease activity in ulcerative colitis. J. Crohn’s Colitis. 8:1378–1391. 10.1016/j.crohns.2014.04.003 24793818 PMC4216648

[bib70] Krishnamoorthy, M., F.H.M. Buhari, T. Zhao, P.M. Brauer, K. Burrows, E.Y. Cao, V. Moxley-Paquette, A. Mortha, J.C. Zuniga-Pflucker, and B. Treanor. 2018. The ion channel TRPM7 is required for B cell lymphopoiesis. Sci. Signal. 11:eaan2693. 10.1126/scisignal.aan2693 29871911

[bib71] Kulkarni, S., J. Ganz, J. Bayrer, L. Becker, M. Bogunovic, and M. Rao. 2018. Advances in enteric neurobiology: The “brain” in the gut in health and disease. J. Neurosci. 38:9346–9354. 10.1523/JNEUROSCI.1663-18.2018 30381426 PMC6209840

[bib72] Kun, J., I. Szitter, A. Kemeny, A. Perkecz, L. Kereskai, K. Pohoczky, A. Vincze, S. Godi, I. Szabo, J. Szolcsanyi, . 2014. Upregulation of the transient receptor potential ankyrin 1 ion channel in the inflamed human and mouse colon and its protective roles. PLoS One. 9:e108164. 10.1371/journal.pone.0108164 25265225 PMC4180273

[bib73] Lai, N.Y., K. Mills, and I.M. Chiu. 2017. Sensory neuron regulation of gastrointestinal inflammation and bacterial host defence. J. Intern. Med. 282:5–23. 10.1111/joim.12591 28155242 PMC5474171

[bib74] Lee, J., T. Yamamoto, H. Kuramoto, and M. Kadowaki. 2012. TRPV1 expressing extrinsic primary sensory neurons play a protective role in mouse oxazolone-induced colitis. Auton. Neurosci. 166:72–76. 10.1016/j.autneu.2011.07.008 21855422

[bib75] Lei, W., and Z. Duan. 2021. Advances in the treatment of cholinergic anti-inflammatory pathways in gastrointestinal diseases by electrical stimulation of vagus nerve. Digestion. 102:128–138. 10.1159/000504474 31786570

[bib76] Letavic, M.A., B.M. Savall, B.D. Allison, L. Aluisio, J.I. Andres, M. De Angelis, H. Ao, D.A. Beauchamp, P. Bonaventure, S. Bryant, . 2017. 4-Methyl-6, 7-dihydro-4H-triazolo[4, 5-c]pyridine-Based P2X7 receptor antagonists: Optimization of pharmacokinetic properties leading to the identification of a clinical candidate. J. Med. Chem. 60:4559–4572. 10.1021/acs.jmedchem.7b00408 28493698

[bib77] Letizia, M., Y.H. Wang, U. Kaufmann, L. Gerbeth, A. Sand, M. Brunkhorst, P. Weidner, J.F. Ziegler, C. Bottcher, S. Schlickeiser, . 2022. Store-operated calcium entry controls innate and adaptive immune cell function in inflammatory bowel disease. EMBO Mol. Med. 14:e15687. 10.15252/emmm.202215687 PMC9449601 35919953

[bib78] Lewis, R.S. 2001. Calcium signaling mechanisms in T lymphocytes. Annu. Rev. Immunol. 19:497–521. 10.1146/annurev.immunol.19.1.497 11244045

[bib79] Li, X., S. Saitoh, T. Shibata, N. Tanimura, R. Fukui, and K. Miyake. 2015. Mucolipin 1 positively regulates TLR7 responses in dendritic cells by facilitating RNA transportation to lysosomes. Int. Immunol. 27:83–94. 10.1093/intimm/dxu086 25239130

[bib80] Liang, X., J. Xie, H. Liu, R. Zhao, W. Zhang, H. Wang, H. Pan, Y. Zhou, and W. Han. 2022. STIM1 deficiency in intestinal epithelium attenuates colonic inflammation and tumorigenesis by reducing ER stress of goblet cells. Cell. Mol. Gastroenterol. Hepatol. 14:193–217. 10.1016/j.jcmgh.2022.03.007 35367664 PMC9130113

[bib81] Link, T.M., U. Park, B.M. Vonakis, D.M. Raben, M.J. Soloski, and M.J. Caterina. 2010. TRPV2 has a pivotal role in macrophage particle binding and phagocytosis. Nat. Immunol. 11:232–239. 10.1038/ni.1842 20118928 PMC2840267

[bib82] Luo, J., A. Qian, L.K. Oetjen, W. Yu, P. Yang, J. Feng, Z. Xie, S. Liu, S. Yin, D. Dryn, . 2018. TRPV4 Channel Signaling in macrophages promotes gastrointestinal motility via direct effects on smooth muscle cells. Immunity. 49:107–119 e4. 10.1016/j.immuni.2018.04.021 29958798 PMC6051912

[bib83] Macpherson, L.J., A.E. Dubin, M.J. Evans, F. Marr, P.G. Schultz, B.F. Cravatt, and A. Patapoutian. 2007. Noxious compounds activate TRPA1 ion channels through covalent modification of cysteines. Nature. 445:541–545. 10.1038/nature05544 17237762

[bib84] Maniscalco, J.W., and L. Rinaman. 2018. Vagal interoceptive modulation of motivated behavior. Physiology. 33:151–167. 10.1152/physiol.00036.2017 29412062 PMC5899236

[bib85] Marques, C.C., M.T. Castelo-Branco, R.G. Pacheco, F. Buongusto, A. do Rosario Jr, A. Schanaider, R. Coutinho-Silva, and H.S. de Souza. 2014. Prophylactic systemic P2X7 receptor blockade prevents experimental colitis. Biochim. Biophys. Acta. 1842:65–78. 10.1016/j.bbadis.2013.10.012 24184714

[bib86] Mathias, R., and P.Y. von der Weid. 2013. Involvement of the NO-cGMP-K(ATP) channel pathway in the mesenteric lymphatic pump dysfunction observed in the Guinea pig model of TNBS-induced ileitis. Am. J. Physiol. Gastrointest. Liver Physiol. 304:G623–G634. 10.1152/ajpgi.00392.2012 23275612

[bib87] Matsui, M., K. Terasawa, J. Kajikuri, H. Kito, K. Endo, P. Jaikhan, T. Suzuki, and S. Ohya. 2018. Histone deacetylases enhance Ca^2+^-activated K^+^ channel KCa3.1 expression in murine inflammatory CD4^+^ T cells. Int. J. Mol. Sci. 19:2942. 10.3390/ijms19102942 30262728 PMC6213394

[bib88] Matsumoto, K., A. Deguchi, A. Motoyoshi, A. Morita, U. Maebashi, T. Nakamoto, S. Kawanishi, M. Sueyoshi, K. Nishimura, K. Takata, . 2020. Role of transient receptor potential vanilloid subtype 4 in the regulation of azoymethane/dextran sulphate sodium-induced colitis-associated cancer in mice. Eur. J. Pharmacol. 867:172853. 10.1016/j.ejphar.2019.172853 31836532

[bib89] Matsumoto, K., R. Yamaba, K. Inoue, D. Utsumi, T. Tsukahara, K. Amagase, M. Tominaga, and S. Kato. 2018. Transient receptor potential vanilloid 4 channel regulates vascular endothelial permeability during colonic inflammation in dextran sulphate sodium-induced murine colitis. Br. J. Pharmacol. 175:84–99. 10.1111/bph.14072 29053877 PMC5740260

[bib90] Matteoli, G., P.J. Gomez-Pinilla, A. Nemethova, M. Di Giovangiulio, C. Cailotto, S.H. van Bree, K. Michel, K.J. Tracey, M. Schemann, W. Boesmans, . 2014. A distinct vagal anti-inflammatory pathway modulates intestinal muscularis resident macrophages independent of the spleen. Gut. 63:938–948. 10.1136/gutjnl-2013-304676 23929694

[bib91] McCarl, C.A., S. Khalil, J. Ma, M. Oh-hora, M. Yamashita, J. Roether, T. Kawasaki, A. Jairaman, Y. Sasaki, M. Prakriya, and S. Feske. 2010. Store-operated Ca^2+^ entry through ORAI1 is critical for T cell-mediated autoimmunity and allograft rejection. J. Immunol. 185:5845–5858. 10.4049/jimmunol.1001796 20956344 PMC2974040

[bib92] McKemy, D.D., W.M. Neuhausser, and D. Julius. 2002. Identification of a cold receptor reveals a general role for TRP channels in thermosensation. Nature. 416:52–58. 10.1038/nature719 11882888

[bib93] Mei, Y., C. Fang, S. Ding, X. Liu, J. Hu, J. Xu, and Q. Mei. 2019. PAP-1 ameliorates DSS-induced colitis with involvement of NLRP3 inflammasome pathway. Int. Immunopharmacol. 75:105776. 10.1016/j.intimp.2019.105776 31351364

[bib94] Mendu, S.K., M.E. Stremska, M.S. Schappe, E.K. Moser, J.K. Krupa, J.S. Rogers, E.J. Stipes, C.A. Parker, T.J. Braciale, J.S.A. Perry, and B.N. Desai. 2020. Targeting the ion channel TRPM7 promotes the thymic development of regulatory T cells by promoting IL-2 signaling. Sci. Signal. 13:eabb0619. 10.1126/scisignal.abb0619 33293462 PMC7884026

[bib95] Muniz, L.R., C. Knosp, and G. Yeretssian. 2012. Intestinal antimicrobial peptides during homeostasis, infection, and disease. Front. Immunol. 3:310. 10.3389/fimmu.2012.00310 23087688 PMC3466489

[bib96] Nadjsombati, M.S., J.W. McGinty, M.R. Lyons-Cohen, J.B. Jaffe, L. DiPeso, C. Schneider, C.N. Miller, J.L. Pollack, G.A. Nagana Gowda, M.F. Fontana, . 2018. Detection of succinate by intestinal tuft cells triggers a type 2 innate immune circuit. Immunity. 49:33–41.e7. 10.1016/j.immuni.2018.06.016 30021144 PMC6084797

[bib97] Neves, A.R., M.T. Castelo-Branco, V.R. Figliuolo, C. Bernardazzi, F. Buongusto, A. Yoshimoto, H.F. Nanini, C.M. Coutinho, A.J. Carneiro, R. Coutinho-Silva, and H.S. de Souza. 2014. Overexpression of ATP-activated P2X7 receptors in the intestinal mucosa is implicated in the pathogenesis of Crohn’s disease. Inflamm. Bowel Dis. 20:444–457. 10.1097/01.MIB.0000441201.10454.06 24412990

[bib98] Niesler, B., S. Kuerten, I.E. Demir, and K.H. Schafer. 2021. Disorders of the enteric nervous system: A holistic view. Nat. Rev. Gastroenterol. Hepatol. 18:393–410. 10.1038/s41575-020-00385-2 33514916

[bib99] Nullens, S., M. Staessens, C. Peleman, D.M. Schrijvers, S. Malhotra-Kumar, S. Francque, G. Matteoli, G.E. Boeckxstaens, J.G. De Man, and B.Y. De Winter. 2016. Effect of gts-21, an α7 nicotinic acetylcholine receptor agonist, on clp-induced inflammatory, gastrointestinal motility, and colonic permeability changes in mice. Shock. 45:450–459. 10.1097/SHK.0000000000000519 26618987

[bib100] O’Leary, C.E., C. Schneider, and R.M. Locksley. 2019. Tuft cells-systemically dispersed sensory epithelia integrating immune and neural circuitry. Annu. Rev. Immunol. 37:47–72. 10.1146/annurev-immunol-042718-041505 30379593 PMC8352721

[bib101] Oh-Hora, M., N. Komatsu, M. Pishyareh, S. Feske, S. Hori, M. Taniguchi, A. Rao, and H. Takayanagi. 2013. Agonist-selected T cell development requires strong T cell receptor signaling and store-operated calcium entry. Immunity. 38:881–895. 10.1016/j.immuni.2013.02.008 23499491 PMC3669219

[bib102] Ohbori, K., M. Fujiwara, A. Ohishi, K. Nishida, Y. Uozumi, and K. Nagasawa. 2017. Prophylactic oral administration of magnesium ameliorates dextran sulfate sodium-induced colitis in mice through a decrease of colonic accumulation of P2X7 receptor-expressing mast cells. Biol. Pharm. Bull. 40:1071–1077. 10.1248/bpb.b17-00143 28674250

[bib103] Ohya, S., Y. Fukuyo, H. Kito, R. Shibaoka, M. Matsui, H. Niguma, Y. Maeda, H. Yamamura, M. Fujii, K. Kimura, and Y. Imaizumi. 2014. Upregulation of KCa3.1 K^+^ channel in mesenteric lymph node CD4^+^ T lymphocytes from a mouse model of dextran sodium sulfate-induced inflammatory bowel disease. Am. J. Physiol. Gastrointest. Liver Physiol. 306:G873–G885. 10.1152/ajpgi.00156.2013 24674776

[bib104] Parenti, A., F. De Logu, P. Geppetti, and S. Benemei. 2016. What is the evidence for the role of TRP channels in inflammatory and immune cells? Br. J. Pharmacol. 173:953–969. 10.1111/bph.13392 26603538 PMC5341240

[bib105] Park, Y.J., S.A. Yoo, M. Kim, and W.U. Kim. 2020. The role of calcium-calcineurin-NFAT signaling pathway in health and autoimmune diseases. Front. Immunol. 11:195. 10.3389/fimmu.2020.00195 32210952 PMC7075805

[bib106] Phillips, R.J., and T.L. Powley. 2007. Innervation of the gastrointestinal tract: Patterns of aging. Auton. Neurosci. 136:1–19. 10.1016/j.autneu.2007.04.005 17537681 PMC2045700

[bib107] Populin, L., M.J. Stebbing, and J.B. Furness. 2021. Neuronal regulation of the gut immune system and neuromodulation for treating inflammatory bowel disease. FASEB Bioadv. 3:953–966. 10.1096/fba.2021-00070 34761177 PMC8565205

[bib108] Proietti, M., V. Cornacchione, T. Rezzonico Jost, A. Romagnani, C.E. Faliti, L. Perruzza, R. Rigoni, E. Radaelli, F. Caprioli, S. Preziuso, . 2014. ATP-gated ionotropic P2X7 receptor controls follicular T helper cell numbers in Peyer’s patches to promote host-microbiota mutualism. Immunity. 41:789–801. 10.1016/j.immuni.2014.10.010 25464855

[bib109] Pugliese, D., A. Armuzzi, F. Castri, R. Benvenuto, A. Mangoni, L. Guidi, A. Gasbarrini, G.L. Rapaccini, F.I. Wolf, and V. Trapani. 2020. TRPM7 is overexpressed in human IBD-related and sporadic colorectal cancer and correlates with tumor grade. Dig. Liver Dis. 52:1188–1194. 10.1016/j.dld.2020.05.027 32505565

[bib110] Quan, J.H., R. Huang, Z. Wang, S. Huang, I.W. Choi, Y. Zhou, Y.H. Lee, and J.Q. Chu. 2018. P2X7 receptor mediates NLRP3-dependent IL-1β secretion and parasite proliferation in *Toxoplasma gondii*-infected human small intestinal epithelial cells. Parasit. Vectors. 11:1. 10.1186/s13071-017-2573-y 29291748 PMC5748956

[bib111] Ramachandran, R., E. Hyun, L. Zhao, T.K. Lapointe, K. Chapman, C.L. Hirota, S. Ghosh, D.D. McKemy, N. Vergnolle, P.L. Beck, . 2013. TRPM8 activation attenuates inflammatory responses in mouse models of colitis. Proc. Natl. Acad. Sci. USA. 110:7476–7481. 10.1073/pnas.1217431110 23596210 PMC3645521

[bib112] Ramirez, V.T., J. Sladek, D.R. Godinez, K.M. Rude, P. Chicco, K. Murray, I. Brust-Mascher, M.G. Gareau, and C. Reardon. 2020. Sensory nociceptive neurons contribute to host protection during enteric infection with Citrobacter rodentium. J. Infect. Dis. 221:1978–1988. 10.1093/infdis/jiaa014 31960920 PMC7289549

[bib113] Rana, M., Y. Fei-Bloom, M. Son, A. La Bella, M. Ochani, Y.A. Levine, P.Y. Chiu, P. Wang, S.S. Chavan, B.T. Volpe, . 2018. Constitutive vagus nerve activation modulates immune suppression in sepsis survivors. Front. Immunol. 9:2032. 10.3389/fimmu.2018.02032 30237803 PMC6135874

[bib114] Rizopoulos, T., H. Papadaki-Petrou, and M. Assimakopoulou. 2018. Expression profiling of the transient receptor potential vanilloid (TRPV) channels 1, 2, 3 and 4 in mucosal epithelium of human ulcerative colitis. Cells. 7:61. 10.3390/cells7060061 29914124 PMC6025154

[bib115] Romagnani, A., V. Vettore, T. Rezzonico-Jost, S. Hampe, E. Rottoli, W. Nadolni, M. Perotti, M.A. Meier, C. Hermanns, S. Geiger, . 2017. TRPM7 kinase activity is essential for T cell colonization and alloreactivity in the gut. Nat. Commun. 8:1917. 10.1038/s41467-017-01960-z 29203869 PMC5714948

[bib116] Rueda Ruzafa, L., J.L. Cedillo, and A.J. Hone. 2021. Nicotinic acetylcholine receptor involvement in inflammatory bowel disease and interactions with gut microbiota. Int. J. Environ. Res. Public Health. 18:1189. 10.3390/ijerph18031189 33572734 PMC7908252

[bib117] Salaga, M., L.V. Blomster, A. Piechota-Polańczyk, M. Zielińska, D. Jacenik, A.I. Cygankiewicz, W.M. Krajewska, J.D. Mikkelsen, and J. Fichna. 2016. Encenicline, an alpha7 nicotinic acetylcholine receptor partial agonist, reduces immune cell infiltration in the colon and improves experimental colitis in mice. J. Pharmacol. Exp. Ther. 356:157–169. 10.1124/jpet.115.228205 26462538

[bib118] Samie, M., X. Wang, X. Zhang, A. Goschka, X. Li, X. Cheng, E. Gregg, M. Azar, Y. Zhuo, A.G. Garrity, . 2013. A TRP channel in the lysosome regulates large particle phagocytosis via focal exocytosis. Dev. Cell. 26:511–524. 10.1016/j.devcel.2013.08.003 23993788 PMC3794471

[bib119] Schappe, M.S., K. Szteyn, M.E. Stremska, S.K. Mendu, T.K. Downs, P.V. Seegren, M.A. Mahoney, S. Dixit, J.K. Krupa, E.J. Stipes, . 2018. Chanzyme TRPM7 mediates the Ca^2+^ influx essential for lipopolysaccharide-induced toll-like receptor 4 endocytosis and macrophage activation. Immunity. 48:59–74.e5. 10.1016/j.immuni.2017.11.026 29343440 PMC5783319

[bib120] Schenk, U., M. Frascoli, M. Proietti, R. Geffers, E. Traggiai, J. Buer, C. Ricordi, A.M. Westendorf, and F. Grassi. 2011. ATP inhibits the generation and function of regulatory T cells through the activation of purinergic P2X receptors. Sci. Signal. 4:ra12. 10.1126/scisignal.2001270 21364186

[bib121] Schenk, U., A.M. Westendorf, E. Radaelli, A. Casati, M. Ferro, M. Fumagalli, C. Verderio, J. Buer, E. Scanziani, and F. Grassi. 2008. Purinergic control of T cell activation by ATP released through pannexin-1 hemichannels. Sci. Signal. 1:ra6. 10.1126/scisignal.1160583 18827222

[bib122] Schneider, C., C.E. O’Leary, J. von Moltke, H.E. Liang, Q.Y. Ang, P.J. Turnbaugh, S. Radhakrishnan, M. Pellizzon, A. Ma, and R.M. Locksley. 2018. A metabolite-triggered tuft cell-ILC2 circuit drives small intestinal remodeling. Cell. 174:271–284.e14. 10.1016/j.cell.2018.05.014 29887373 PMC6046262

[bib123] Schwarz, A., E. Tutsch, B. Ludwig, E.C. Schwarz, A. Stallmach, and M. Hoth. 2004. Ca^2+^ signaling in identified T-lymphocytes from human intestinal mucosa. Relation to hyporeactivity, proliferation, and inflammatory bowel disease. J. Biol. Chem. 279:5641–5647. 10.1074/jbc.M309317200 14585840

[bib124] Shimokawa, C., T. Kanaya, M. Hachisuka, K. Ishiwata, H. Hisaeda, Y. Kurashima, H. Kiyono, T. Yoshimoto, T. Kaisho, and H. Ohno. 2017. Mast cells are crucial for induction of group 2 innate lymphoid cells and clearance of helminth infections. Immunity. 46:863–874.e4. 10.1016/j.immuni.2017.04.017 28514691

[bib125] Simms, L.A., J.D. Doecke, R.L. Roberts, E.V. Fowler, Z.Z. Zhao, M.A. McGuckin, N. Huang, N.K. Hayward, P.M. Webb, D.C. Whiteman, . 2010. KCNN4 gene variant is associated with ileal Crohn’s Disease in the Australian and New Zealand population. Am. J. Gastroenterol. 105:2209–2217. 10.1038/ajg.2010.161 20407432

[bib126] Smith-Edwards, K.M., B.S. Edwards, C.M. Wright, S. Schneider, K.A. Meerschaert, L.L. Ejoh, S.A. Najjar, M.J. Howard, K.M. Albers, R.O. Heuckeroth, and B.M. Davis. 2021. Sympathetic input to multiple cell types in mouse and human colon produces region-specific responses. Gastroenterology. 160:1208–1223.e4. 10.1053/j.gastro.2020.09.030 32980343 PMC7956113

[bib127] Snoek, S.A., M.I. Verstege, E.P. van der Zanden, N. Deeks, D.C. Bulmer, M. Skynner, K. Lee, A.A. Te Velde, G.E. Boeckxstaens, and W.J. de Jonge. 2010. Selective α7 nicotinic acetylcholine receptor agonists worsen disease in experimental colitis. Br. J. Pharmacol. 160:322–333. 10.1111/j.1476-5381.2010.00699.x 20423343 PMC2874854

[bib128] Spencer, N.J., and H. Hu. 2020. Enteric nervous system: Sensory transduction, neural circuits and gastrointestinal motility. Nat. Rev. Gastroenterol. Hepatol. 17:338–351. 10.1038/s41575-020-0271-2 32152479 PMC7474470

[bib129] Stakenborg, N., E. Labeeuw, P.J. Gomez-Pinilla, S. De Schepper, R. Aerts, G. Goverse, G. Farro, I. Appeltans, E. Meroni, M. Stakenborg, . 2019. Preoperative administration of the 5-HT4 receptor agonist prucalopride reduces intestinal inflammation and shortens postoperative ileus via cholinergic enteric neurons. Gut. 68:1406–1416. 10.1136/gutjnl-2018-317263 30472681 PMC6691854

[bib130] Stein, A.T., C.A. Ufret-Vincenty, L. Hua, L.F. Santana, and S.E. Gordon. 2006. Phosphoinositide 3-kinase binds to TRPV1 and mediates NGF-stimulated TRPV1 trafficking to the plasma membrane. J. Gen. Physiol. 128:509–522. 10.1085/jgp.200609576 17074976 PMC2151588

[bib131] Strobaek, D., D.T. Brown, D.P. Jenkins, Y.J. Chen, N. Coleman, Y. Ando, P. Chiu, S. Jorgensen, J. Demnitz, H. Wulff, and P. Christophersen. 2013. NS6180, a new K_Ca_3.1 channel inhibitor prevents T-cell activation and inflammation in a rat model of inflammatory bowel disease. Br. J. Pharmacol. 168:432–444. 10.1111/j.1476-5381.2012.02143.x 22891655 PMC3572569

[bib132] Suss, C., L. Broncy, K. Pollinger, C. Kunst, K. Gulow, M. Muller, and G. Wolfel. 2020. KCNN4 expression is elevated in inflammatory bowel disease: This might Be a novel marker and therapeutic option targeting potassium channels. J. Gastrointestin. Liver Dis. 29:539–547. 10.15403/jgld-903 33331347

[bib133] Szitter, I., G. Pozsgai, K. Sandor, K. Elekes, A. Kemeny, A. Perkecz, J. Szolcsanyi, Z. Helyes, and E. Pinter. 2010. The role of transient receptor potential vanilloid 1 (TRPV1) receptors in dextran sulfate-induced colitis in mice. J. Mol. Neurosci. 42:80–88. 10.1007/s12031-010-9366-5 20411352

[bib134] Takiishi, T., C.I.M. Fenero, and N.O.S. Camara. 2017. Intestinal barrier and gut microbiota: Shaping our immune responses throughout life. Tissue Barriers. 5:e1373208. 10.1080/21688370.2017.1373208 28956703 PMC5788425

[bib135] Tani, H., B. Li, T. Kusu, R. Okumura, J. Nishimura, D. Okuzaki, D. Motooka, S. Arakawa, A. Mori, T. Yoshihara, . 2021. The ATP-hydrolyzing ectoenzyme E-NTPD8 attenuates colitis through modulation of P2X4 receptor-dependent metabolism in myeloid cells. Proc. Natl. Acad. Sci. USA. 118:e2100594118. 10.1073/pnas.2100594118 34548395 PMC8488689

[bib136] Tian, C., L. Du, Y. Zhou, and M. Li. 2016. Store-operated CRAC channel inhibitors: Opportunities and challenges. Future Med. Chem. 8:817–832. 10.4155/fmc-2016-0024 27149324 PMC5558521

[bib137] Tilg, H., and T.E. Adolph. 2017. Beyond digestion: The pancreas shapes intestinal microbiota and immunity. Cell Metabol. 25:495–496. 10.1016/j.cmet.2017.02.018 28273472

[bib138] Toft-Bertelsen, T.L., and N. MacAulay. 2021. TRPing on cell swelling - TRPV4 senses it. Front. Immunol. 12:730982. 10.3389/fimmu.2021.730982 34616399 PMC8488219

[bib139] Tsuchida, Y., F. Hatao, M. Fujisawa, T. Murata, M. Kaminishi, Y. Seto, M. Hori, and H. Ozaki. 2011. Neuronal stimulation with 5-hydroxytryptamine 4 receptor induces anti-inflammatory actions via α7nACh receptors on muscularis macrophages associated with postoperative ileus. Gut. 60:638–647. 10.1136/gut.2010.227546 21115544 PMC3071096

[bib140] Turner, J.R. 2009. Intestinal mucosal barrier function in health and disease. Nat. Rev. Immunol. 9:799–809. 10.1038/nri2653 19855405

[bib141] Unterweger, A.L., M.O. Jensen, F. Giordanetto, V. Jogini, A. Ruschher, M. Seuß, P. Winkelmann, L. Koletzko, D.E. Shaw, M. Siebeck, . 2021. Suppressing Kv1.3 Ion Channel activity with a novel small molecule inhibitor ameliorates inflammation in a humanised mouse model of ulcerative colitis. J. Crohn’s Colitis. 15:1943–1958. 10.1093/ecco-jcc/jjab078 33891001 PMC8575044

[bib142] Utsumi, D., K. Matsumoto, T. Tsukahara, K. Amagase, M. Tominaga, and S. Kato. 2018. Transient receptor potential vanilloid 1 and transient receptor potential ankyrin 1 contribute to the progression of colonic inflammation in dextran sulfate sodium-induced colitis in mice: Links to calcitonin gene-related peptide and substance P. J. Pharmacol. Sci. 136:121–132. 10.1016/j.jphs.2017.12.012 29478714

[bib143] Vaeth, M., S. Kahlfuss, and S. Feske. 2020. CRAC channels and calcium signaling in T cell-mediated immunity. Trends Immunol. 41:878–901. 10.1016/j.it.2020.06.012 32711944 PMC7985820

[bib144] Van Der Zanden, E.P., G.E. Boeckxstaens, and W.J. de Jonge. 2009a. The vagus nerve as a modulator of intestinal inflammation. Neurogastroenterol. Motil. 21:6–17. 10.1111/j.1365-2982.2008.01252.x 19140954

[bib145] van der Zanden, E.P., S.A. Snoek, S.E. Heinsbroek, O.I. Stanisor, C. Verseijden, G.E. Boeckxstaens, M.P. Peppelenbosch, D.R. Greaves, S. Gordon, and W.J. De Jonge. 2009b. Vagus nerve activity augments intestinal macrophage phagocytosis via nicotinic acetylcholine receptor α4β2. Gastroenterology. 137:1029–1039. 10.1053/j.gastro.2009.04.057 19427310

[bib146] Vig, M., and J.P. Kinet. 2009. Calcium signaling in immune cells. Nat. Immunol. 10:21–27. 10.1038/ni.f.220 19088738 PMC2877033

[bib147] Wang, H., J.P.P. Foong, N.L. Harris, and J.C. Bornstein. 2022. Enteric neuroimmune interactions coordinate intestinal responses in health and disease. Mucosal Immunol. 15:27–39. 10.1038/s41385-021-00443-1 34471248 PMC8732275

[bib148] Wang, H., M. Yu, M. Ochani, C.A. Amella, M. Tanovic, S. Susarla, J.H. Li, H. Wang, H. Yang, L. Ulloa, . 2003. Nicotinic acetylcholine receptor alpha7 subunit is an essential regulator of inflammation. Nature. 421:384–388. 10.1038/nature01339 12508119

[bib149] Weidinger, C., P.J. Shaw, and S. Feske. 2013. STIM1 and STIM2-mediated Ca^2+^ influx regulates antitumour immunity by CD8^+^ T cells. EMBO Mol. Med. 5:1311–1321. 10.1002/emmm.201302989 23922331 PMC3799488

[bib150] Wood, J.D. 2016. Enteric neurobiology: Discoveries and directions. Adv. Exp. Med. Biol. 891:175–191. 10.1007/978-3-319-27592-5_17 27379645

[bib151] Wu, L.J., T.B. Sweet, and D.E. Clapham. 2010. International Union of Basic and Clinical Pharmacology. LXXVI. Current progress in the mammalian TRP ion channel family. Pharmacol. Rev. 62:381–404. 10.1124/pr.110.002725 20716668 PMC2964900

[bib152] Wulff, H., and N.A. Castle. 2010. Therapeutic potential of KCa3.1 blockers: Recent advances and promising trends. Expert Rev. Clin. Pharmacol. 3:385–396. 10.1586/ecp.10.11 22111618 PMC3347644

[bib153] Xiao, J., G. Zhang, S. Gao, J. Shen, H. Feng, Z. He, and C. Xu. 2020. Combined administration of SHP2 inhibitor SHP099 and the α7nAChR agonist PNU282987 protect mice against DSS-induced colitis. Mol. Med. Rep. 22:2235–2244. 10.3892/mmr.2020.11324 32705242 PMC7411392

[bib154] Xie, Z., and H. Hu. 2018. TRP channels as drug targets to relieve itch. Pharmaceuticals. 11:100. 10.3390/ph11040100 30301231 PMC6316386

[bib155] Yamamoto, S., S. Shimizu, S. Kiyonaka, N. Takahashi, T. Wajima, Y. Hara, T. Negoro, T. Hiroi, Y. Kiuchi, T. Okada, . 2008. TRPM2-mediated Ca^2+^ influx induces chemokine production in monocytes that aggravates inflammatory neutrophil infiltration. Nat. Med. 14:738–747. 10.1038/nm1758 18542050 PMC2789807

[bib156] Yamamoto, T., T. Kodama, J. Lee, N. Utsunomiya, S. Hayashi, H. Sakamoto, H. Kuramoto, and M. Kadowaki. 2014. Anti-allergic role of cholinergic neuronal pathway via α7 nicotinic ACh receptors on mucosal mast cells in a murine food allergy model. PLoS One. 9:e85888. 10.1371/journal.pone.0085888 24454942 PMC3894205

[bib157] Yang, N.N., J.W. Yang, Y. Ye, J. Huang, L. Wang, Y. Wang, X.T. Su, Y. Lin, F.T. Yu, S.M. Ma, . 2021. Electroacupuncture ameliorates intestinal inflammation by activating α7nAChR-mediated JAK2/STAT3 signaling pathway in postoperative ileus. Theranostics. 11:4078–4089. 10.7150/thno.52574 33754049 PMC7977469

[bib158] Yelshanskaya, M.V., K.D. Nadezhdin, M.G. Kurnikova, and A.I. Sobolevsky. 2021. Structure and function of the calcium-selective TRP channel TRPV6. J. Physiol. 599:2673–2697. 10.1113/JP279024 32073143 PMC7689878

[bib159] Zhang, S., T. Al-Maghout, H. Cao, L. Pelzl, M.S. Salker, M. Veldhoen, A. Cheng, F. Lang, and Y. Singh. 2019. Gut bacterial metabolite urolithin A (ua) mitigates Ca^2+^ entry in T cells by regulating miR-10a-5p. Front. Immunol. 10:1737. 10.3389/fimmu.2019.01737 31417547 PMC6685097

[bib160] Zhang, X., B. Lei, Y. Yuan, L. Zhang, L. Hu, S. Jin, B. Kang, X. Liao, W. Sun, F. Xu, . 2020. Brain control of humoral immune responses amenable to behavioural modulation. Nature. 581:204–208. 10.1038/s41586-020-2235-7 32405000

[bib161] Zhao, J., J.V. Lin King, C.E. Paulsen, Y. Cheng, and D. Julius. 2020. Irritant-evoked activation and calcium modulation of the TRPA1 receptor. Nature. 585:141–145. 10.1038/s41586-020-2480-9 32641835 PMC7483980

[bib162] Zheng, W., H. Song, Z. Luo, H. Wu, L. Chen, Y. Wang, H. Cui, Y. Zhang, B. Wang, W. Li, . 2021. Acetylcholine ameliorates colitis by promoting IL-10 secretion of monocytic myeloid-derived suppressor cells through the nAChR/ERK pathway. Proc. Natl. Acad. Sci. USA. 118:e2017762118. 10.1073/pnas.2017762118 33836585 PMC7980392

[bib163] Zhou, L., L.F. Zheng, X.L. Zhang, Z.Y. Wang, Y.S. Yao, X.L. Xiu, C.Z. Liu, Y. Zhang, X.Y. Feng, and J.X. Zhu. 2021. Activation of α7nAChR protects against gastric inflammation and dysmotility in Parkinson’s disease rats. Front. Pharmacol. 12:793374. 10.3389/fphar.2021.793374 34880768 PMC8646045

